# Critical transition of soil microbial diversity and composition triggered by plant rhizosphere effects

**DOI:** 10.3389/fpls.2023.1252821

**Published:** 2023-11-10

**Authors:** Xianheng Fu, Yu Huang, Qi Fu, Yingbo Qiu, Jiayi Zhao, Jiaxin Li, Xicun Wu, Yihang Yang, Hongen Liu, Xian Yang, Huaihai Chen

**Affiliations:** State Key Laboratory of Biocontrol, School of Ecology, Sun Yat-sen University, Shenzhen, Guangdong, China

**Keywords:** rhizosphere, meta-analysis, r-strategists, K-strategists, microbiome

## Abstract

Over the years, microbial community composition in the rhizosphere has been extensively studied as the most fascinating topic in microbial ecology. In general, plants affect soil microbiota through rhizodeposits and changes in abiotic conditions. However, a consensus on the response of microbiota traits to the rhizosphere and bulk soils in various ecosystems worldwide regarding community diversity and structure has not been reached yet. Here, we conducted a meta-analysis of 101 studies to investigate the microbial community changes between the rhizosphere and bulk soils across various plant species (maize, rice, vegetables, other crops, herbaceous, and woody plants). Our results showed that across all plant species, plant rhizosphere effects tended to reduce the rhizosphere soil pH, especially in neutral or slightly alkaline soils. Beta-diversity of bacterial community was significantly separated between into rhizosphere and bulk soils. Moreover, r-strategists and copiotrophs (e.g. Proteobacteria and Bacteroidetes) enriched by 24-27% in the rhizosphere across all plant species, while K-strategists and oligotrophic (e.g. Acidobacteria, Gemmatimonadete, Nitrospirae, and Planctomycetes) decreased by 15-42% in the rhizosphere. Actinobacteria, Firmicutes, and Chloroflexi are also depleted by in the plant rhizosphere compared with the bulk soil by 7-14%. The Actinobacteria exhibited consistently negative effect sizes across all plant species, except for maize and vegetables. In Firmicutes, both herbaceous and woody plants showed negative responses to rhizosphere effects, but those in maize and rice were contrarily enriched in the rhizosphere. With regards to Chloroflexi, apart from herbaceous plants showing a positive effect size, the plant rhizosphere effects were consistently negative across all other plant types. Verrucomicrobia exhibited a significantly positive effect size in maize, whereas herbaceous plants displayed a negative effect size in the rhizosphere. Overall, our meta-analysis exhibited significant changes in microbial community structure and diversity responding to the plant rhizosphere effects depending on plant species, further suggesting the importance of plant rhizosphere to environmental changes influencing plants and subsequently their controls over the rhizosphere microbiota related to nutrient cycling and soil health.

## 1 Introduction

The rhizosphere is the soil volume around the root with a rich diversity of microorganisms, which is strongly affected by root functioning (Philippot et al., 2013; Kuzyakov and Razavi, 2019; Qu et al., 2020). The individual and interconnected processes occurring in the rhizosphere have been extensively characterized, encompassing exudate release, nutrient acquisition, and water uptakes (Philippot et al., 2013; Sasse et al., 2018; Kuzyakov and Razavi, 2019). These processes have contributed to the development of a distinct microbial community structure in the rhizosphere compared to the bulk soil, commonly referred to as the rhizosphere effect (Aira et al., 2010; Fan et al., 2017; Sasse et al., 2018; Ling et al., 2022). The rhizosphere microbiome can exert significant influences on plant health, nutrition, and growth (Berendsen et al., 2012; Philippot et al., 2013; Finkel et al., 2017). Plants benefit from rhizosphere microorganisms to help acquire nutrients and suppress pathogenic invasions (Bulgarelli et al., 2013; Poole, 2017; Ling et al., 2022). For example, plant growth-promoting rhizobacteria (PGPR) promotes plant growth through a wide range of mechanisms, which is beneficial for the sustainability of agriculture as the biofertilizers and biopesticides (Pii et al., 2015). Similarly, legumes require rhizobia and mycorrhizal fungi to improve plant productivity and N_2_ fixation (van der Heijden et al., 2008; Kaschuk et al., 2010).

Although genotypes, root architecture, and growth stages tend to affect the plant recruits relatively distinct rhizobacterial communities (Aira et al., 2010; Li et al., 2014), plant itself exerts a highly selective effect to shape the microbial community composition in the rhizosphere, so the community composition can be greatly similar across different environments (Marschner et al., 2004; Costa et al., 2006; Berg and Smalla, 2009; Ling et al., 2022). In addition, soils covered with vegetation, as one of the sources of atmospheric CO_2_, may strongly contribute to the CO_2_ efflux by root and rhizomicrobial respiration (Kuzyakov, 2006; Werth and Kuzyakov, 2008; Trivedi et al., 2013). The distinct rhizomicrobial respiration processes (microbial respiration or respiration by heterotrophs), regulating soil organic matter (SOM) decomposition, was identified as one of the important fine-scale components of the global carbon (C) cycle (Cheng, 1999; Kuzyakov, 2006; Huo et al., 2017; Jackson et al., 2019). The microbial community control over C cycling in the rhizosphere has been extensively studied (Kuzyakov, 2002; Schimel and Schaeffer, 2012; Schindlbacher et al., 2015; Kumar et al., 2016; Hunninghaus et al., 2019; Semenov et al., 2019). Notably, some microbiota exhibited strong resistance to perturbations, while other specific microorganisms respond rapidly to changing environmental conditions (Jiang et al., 2017). This caused a weak but measurable effect on the rhizosphere microbial community even within a single plant species (Bokulich et al., 2014). Therefore, comprehending the taxonomic profiles of microbial communities in the rhizosphere and bulk soil is critical to understand the microbial functions to support plant growth and manage C cycling in the rhizosphere. However, the information in the rhizosphere and bulk soils with respect to the taxonomic profiles of microbial communities remains largely unexplored.

Both plant species and soil properties affect the diversity and structure of rhizosphere microbial community (Garbeva et al., 2008; Jiang et al., 2017; Vorholt et al., 2017). The impact of soil characteristics on rhizosphere microbial community is as significant as that of the plant itself (Marschner et al., 2004; Fan et al., 2018). In general, plant root systems alter the rhizosphere pH by releasing H^+^ or OH^−^, and affecting the equilibrium between cations and anions at the root-soil interface (Hinsinger et al., 2003; Kuzyakov and Razavi, 2019). The pH of the soil is a key factor in determining changes in the structure and diversity of the microbial community (Tripathi et al., 2018; Kuzyakov and Razavi, 2019; Lopes et al., 2021). As previous studies reported that soil pH was the best predictor of soil microbial community diversity (Fierer and Jackson, 2006). Therefore, investigating rhizosphere microbiomes is critical for establishing a more complete knowledge of the role of soil pH on microbial ecology. However, information is lacking on the association of rhizosphere soil pH with the plant species.

Recently, sequencing and phylogenetic analysis of cultivation-independent 16S rRNA genes provided the foundation for modern studies of microorganisms living in the soil (Lundberg et al., 2012; Fan et al., 2017; Fan et al., 2018; Ling et al., 2022). High-throughput sequencing enables quantitative insights into microbial community diversity and structure in high resolution (Singer et al., 2016). Compared to traditional microbial community analyses, high-throughput sequencing is known for its labor efficiency and cost-effectiveness (Reuter et al., 2015). Most studies must be deposited the raw data in a public gene bank, causing a huge and extensive rhizosphere sequencing data set, which has cracked the way to further research into the broad principles of rhizosphere microbiome selection from bulk soils (Ling et al., 2022). Thus, it was urgently needed for a comprehensive study synthesizing previous findings to infer the difference in the microbial community structure between rhizosphere and bulk soils to a wide range of plants and environmental conditions.

Here, we conducted a global meta-analysis of microbial communities in the rhizosphere and bulk soil, with a specific focus on bacteria and fungi due to their high prevalence and the extensive attention they have received in comparison to other members of the community (e.g., archaea, protists, and nematodes, etc.) (Ling et al., 2022). The 16S and ITS rRNA amplicon-based sequencing data were collected from published articles to date. Specifically, our objective was to answer the following questions: (i) how plant rhizosphere affects soil pH and microbial diversity and composition, (ii) to what extent major microbial taxa respond to plant rhizosphere effects, (iii) whether the plant rhizosphere effects on microbial community were dependent on plant species?

## 2 Materials and methods

### 2.1 Data collection

**Figure 1:**
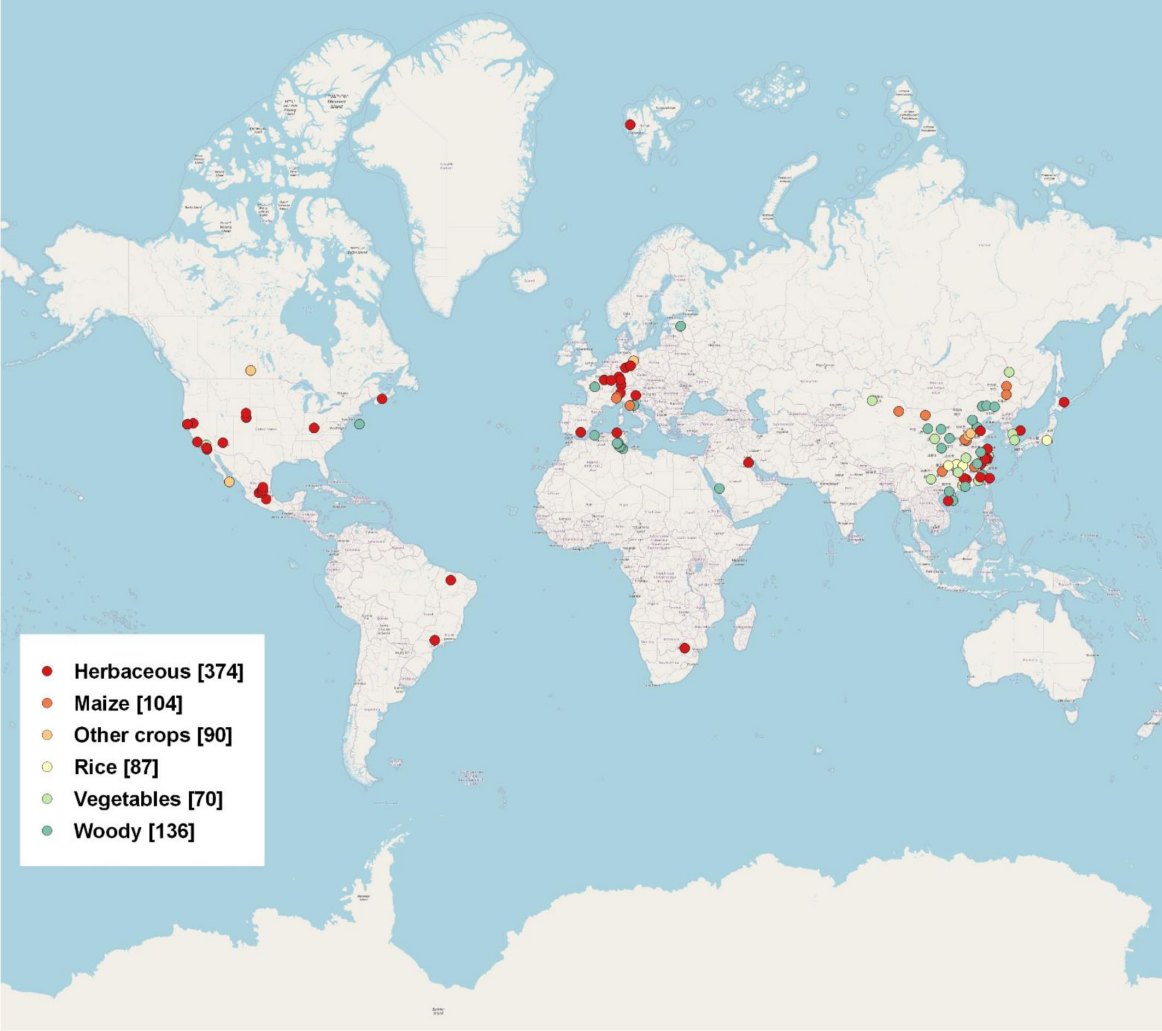
Global distribution of rhizosphere and bulk data used in this meta-analysis, including 861 data points from 101 publications. Six groups of plant species are shown in the legends, with sample sizes for each group given in parentheses.

An extensive literature search was conducted using the Web of Science database (http://apps.webofknowledge.com/). The data was collected from peer-reviewed publications from 2014 to 2021 for our literature survey and review. We search for terms including “rhizosphere”, “bulk”, “fungi”, “bacteria”, “microbial community”, “DNA extraction”, “PCR Amplification”, “16s”, “ITS”, “high-throughput”, “pyrosequencing”, and “Illumina”, etc. in the title, keyword, or abstract. We obtained a total of 861 data points based on 101 publications around the world (**Figure 1**). Detailed information was given in **Table S1**. The data from manipulation experiments conducted in laboratory settings were excluded. We exclusively employed 16S and ITS data, as detailed in **Table S1**, for the purpose of this study. We examined the microbial community structure of bacteria and fungi by analyzing their relative abundance at the phylum level. The phylum level is often employed in rhizosphere and bulk soil research, and findings for the majority of microbial community analyses are commonly based on high-throughput sequencing technique.

The means (M), standard deviations (SD), and sample sizes (n) were obtained from both rhizosphere and bulk soils in each study. If only the standard error (SE) was provided, SD was calculated as SE multiplied by the square root of sample size. Missing standard deviations were estimated using the average coefficient of variation of datasets with known SDs (Chen et al., 2019). Data in tables were directly transferred to our dataset, while data in figures were extracted using Data Thief software, which is specifically designed for retrieving axis-related information from images (B. Tummers, DataThief III. 2006 https://datathief.org/). To ensure the accuracy and quality of the extracted data, only taxa with an average relative abundance > 2% were included. Taxa that were absent in any treatment or replicate of a study were excluded from the data extraction process (Dai et al., 2018).

### 2.2 Meta-analysis

The 16S and ITS data collected in this study were utilized for meta-analysis. The impacts of plant roots on soil microbial compositions were evaluated using the rhizosphere soil against the pairwise bulk soil (Chen et al., 2022). The natural log-transformed response ratio was previously described (Gurevitch et al., 2018):


(1) $$ln\;R=ln(X_{t})-ln(X_{c})$$

Where ln *R* denotes the natural log of response ratio and is defined as the effect size, *X_t_*, and *X_c_* are the mean value of the rhizosphere and bulk soil, respectively, which are directly extracted from data of publications included in this meta-analysis. The ln *R* was further weighted by the pooled variance(*v*):


(2) $$v=\frac{SD_{t}^{2}}{n_{t}X_{t}^{2}}+\frac{SD_{c}^{2}}{n_{c}X_{c}^{2}}$$

where *SD_t_* and *SD_c_* are the SD of rhizosphere and bulk soil, respectively, *n_t_* and *n_c_* are the sample size of the rhizosphere and bulk soil, respectively, which are directly extracted from data of publications included in this meta-analysis. The results obtained from both meta-analysis approaches were identical. We only reported the grand means and bias-corrected 95% CIs of effect sizes calculated by MetaWin because the 95% CIs from this approach enable us to assess the potential publication bias.

The total heterogeneity (Q_T_) of the dataset (i.e. microbial structure and soil pH) was divided into within-group (Q_W_) and between-group (Q_b_) variations. The Q statistic has k-1 degrees of freedom and follows a Chi-square distribution, where k is the number of matched observations between rhizosphere and bulk soils. When the bias-corrected 95% CIs did not overlap zero, the effect size was considered substantially positive or negative at the = 0.05 level (Gurevitch et al., 2018). The lnR (effect size) was transformed and revealed as the percentage variation under rhizosphere relative to bulk soil:


(3) (R−1)×100%

where R denotes the response ratio. In our meta-analysis, the soil pH of rhizosphere was divided into five groups as follows: 5 (pH< 5), 6 (5 ≤ pH<6), 7 (6 ≤ pH<7), 8 (7 ≤ pH<8), and 9 (8 ≤ pH<9). The groups of soil pH were used to examine if there were any significant differences in effect size among the groups. The plant species were divided into maize, rice, vegetables, other crops, herbaceous, and woody plants. The means of effect size between the groups of soil pH or plant species were considered to be significant at α = 0.05 when bias-corrected 95% CIs were non-overlapping.

### 2.3 Statistics

Based on the relative abundance of major fungal and bacterial phylum, we calculated Bray-Curtis dissimilarity for principal coordinate analysis (PCoA). PCoA was performed to visualize the microbial community structure of rhizosphere and bulk soil. Bray-Curtis similarity was calculated to construct triangular pairwise Bray-Curtis similarity matrix using PRIMER 7 (Plymouth Routines in Multivariate Ecological Research Statistical Software, v7.0.13, PRIMER-E Ltd, UK). ANOSIM and ADONIS tests were used to test the cluster significance of samples from the Bray-Curtis similarity distances, which were calculated based on the relative abundance of the major microbial groups. Taxonomic classification was performed using the Silva 16S rRNA and the UNITE fungal ITS reference database in all publications which we obtained. Most statistical analyses were performed in R (v4.0.1; http://www.r-project.org/).

## 3 Results

### 3.1 Soil pH in the rhizosphere and the bulk soil

**Figure 2:**
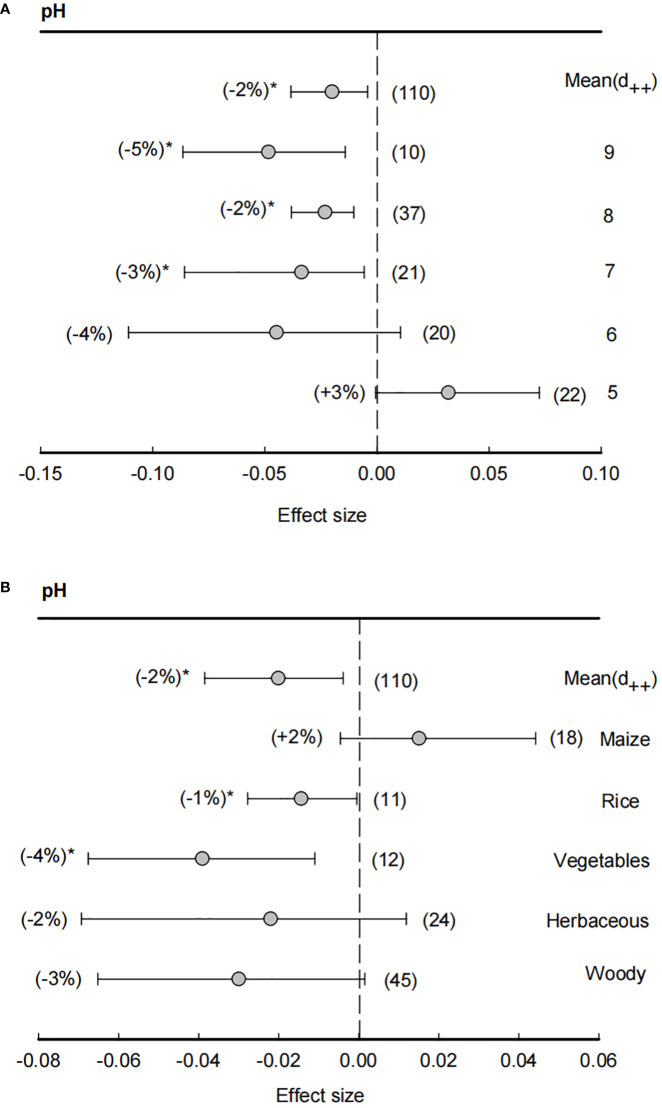
Effect size of soil pH **(A)** and plant species effect on soil pH **(B)**. The bulk soil pH was grouped ranging from 5 (pH< 5), 6 (5 ≤ pH<6), 7 (6 ≤ pH<7), 8 (7 ≤ pH<8) and 9 (8 ≤ pH<9). Plant species including maize, rice, vegetables, herbaceous and woody plants. Data are expressed as the mean effect size (d_++_) with bias-corrected 95% confidence intervals. Percentage changes for means and observation numbers for the category are given in parentheses. Asterisk indicates *P*< 0.05.

Across all studies, the means of effect size of soil pH was -2%, indicating a significantly (*P<*0.05) negative effect in rhizosphere than bulk soils on soil pH (**Figure 2**). In particular, when soil pH > 7, effect sizes of soil pH were significantly (*P<*0.05) lower than zero, showing that rhizosphere effects on soil pH were most in neutral or slightly alkaline soils. However, with a decline of soil pH, the effect sizes of rhizosphere also increased and became positive (+3%) under soil pH = 5, suggesting that bulk soil pH condition was the main factor for the rhizosphere effects on soil pH. Similarly, the effect size of rhizosphere on soil pH was also dependent on plant species, in which the effect sizes in rice (-1%) and vegetables (-4%) were significantly (*P<*0.05) lower than zero, while herbaceous and woody has non-significant effect sizes and maize even had a positive effects size (+2%).

### 3.2 The composition and diversity of soil microbial community

**Figure 3:**
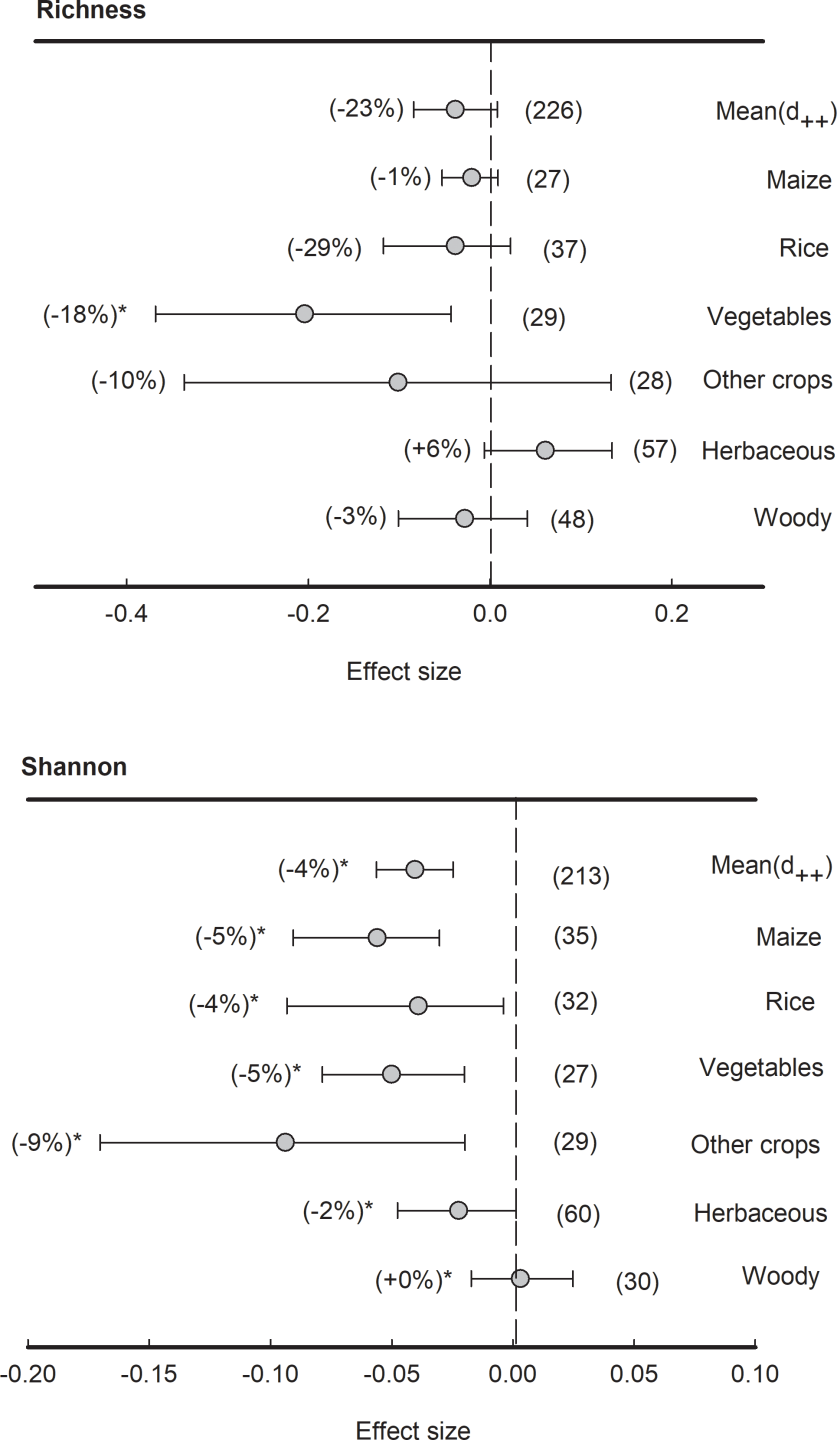
Effects of plant species on bacterial alpha-diversity, i.e., Richness and Shannon indices, grouped by the plant species. Data is expressed as the mean effect size (d_++_) with bias-corrected 95% confidence intervals. Percentage changes for means and observation numbers for the category are given in parentheses. Asterisk indicates *P*< 0.05.

Although the means of effect size of bacterial richness were not significantly different from zero, the effect size of bacterial richness in vegetables was significantly (*P<*0.05) negative (-29%) (**Figure 3**). However, across all plant species, the means of effect size of bacterial Shannon index were significantly (*P<*0.05) negative (-4%). Specifically, the effect sizes of bacterial Shannon index in plant types of maize, rice, vegetables, herbaceous and other crops were significantly (*P<*0.05) lower than zero (-9% to -2%) while those in wood plants were non-significant.

**Figure 4:**
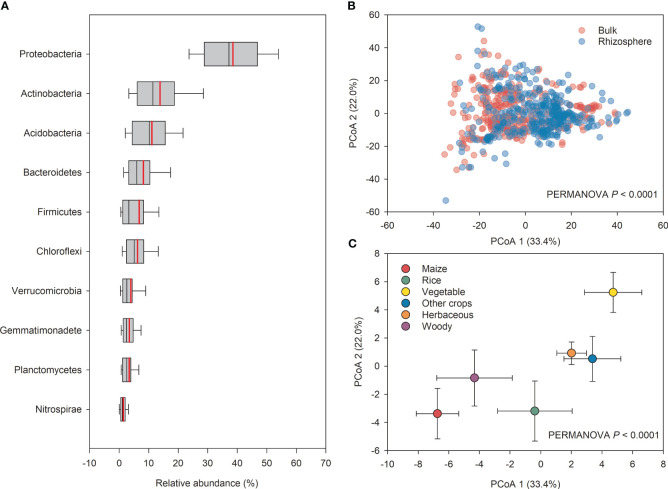
The relative abundance of major bacterial taxas at the phylum levels **(A)**, the principal coordinate analysis (PCoA) of microbial beta-diversity based on Bray-Curtis similarity distances between rhizosphere and bulk soils **(B)** and among plant species **(C)**.

Across all studies, the bacterial communities mainly consist of Proteobacteria (39%), Actinobacteria (14%), Acidobacteria (11%), Bacteroidetes (8%), Firmicutes (7%), Chloroflexi (6%), Verrucomicrobia (4%), Gemmatimonadete (4%), Planctomycetes (3%), and Nitrospirae (1%), with the relative abundance in the descending order (**Figure 4A**). Based on the composition of major phyla found in our study, PCoA of the Bray-Curtis distances was conducted to reveal that the beta-diversity of bacterial community was significantly separated between into rhizosphere and bulk soils (PERMANOVA, *P*< 0.0001) (**Figure 4B**). Specifically, the bacterial community structure of rhizosphere soils was also dependent on plant species (PERMANOVA, *P*< 0.0001) (**Figure 4C**). For example, the bacterial community structure in vegetable rhizosphere was significantly separated from those in maize, rice, and woody plants (PERMANOVA, *P*< 0.0001).

### 3.3 Variation of the phylum in different plant species rhizosphere soil

**Figure 5:**
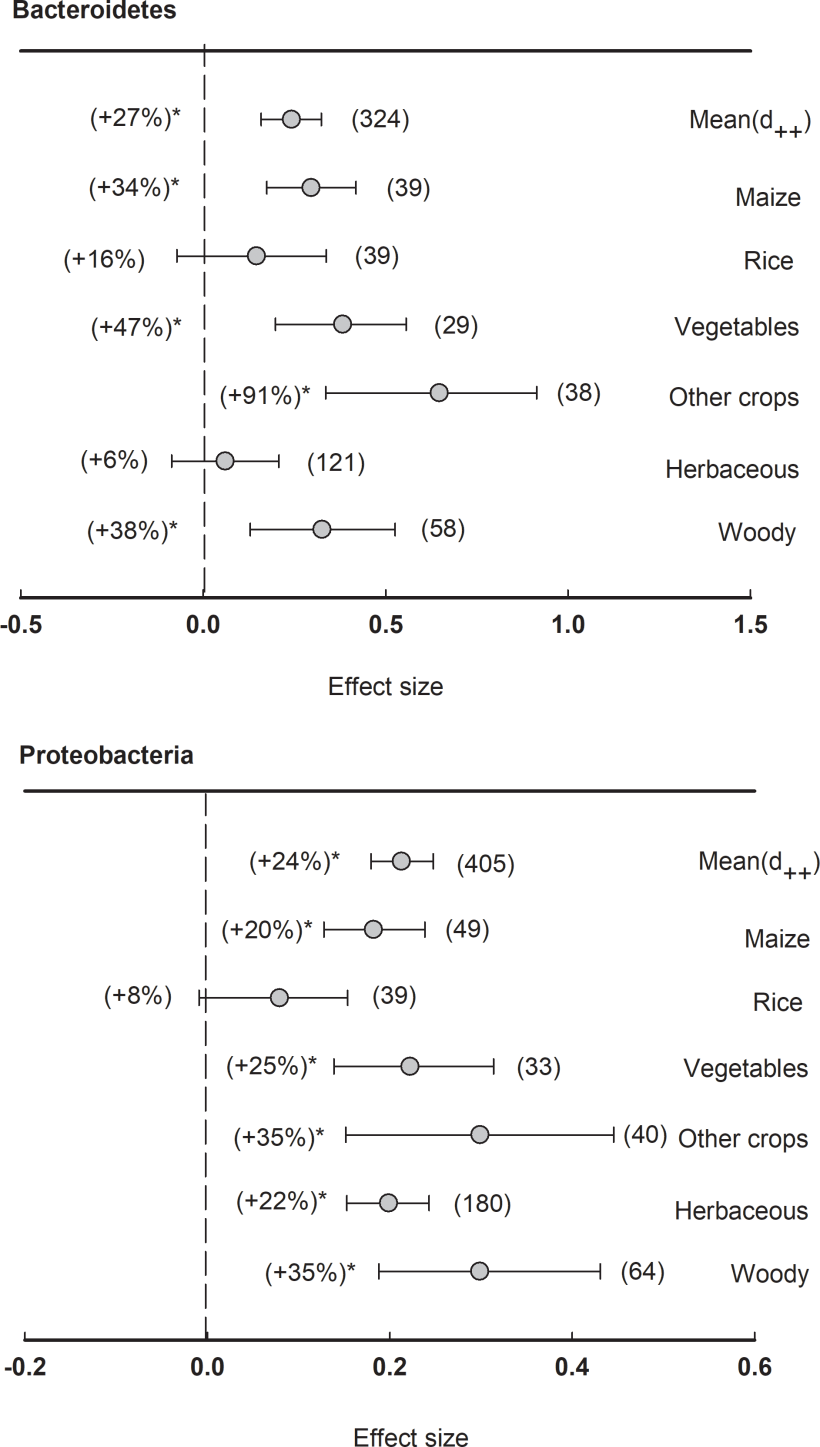
Plant rhizosphere effects on major bacterial phyla (mean proportion > 5%), i.e., Bacteriodetes and Proteobacteria, grouped by the plant species including maize, rice, vegetables, other crops, herbaceous and woody plants. Data is expressed as the mean effect size (d_++_) with bias-corrected 95% confidence intervals. Percentage changes for means and observation numbers for the category are given in parentheses. Asterisk indicates *P*< 0.05.

In total, there were two major phyla showing significantly (*P<*0.05) positive effect size of plant rhizosphere, including Bacteroidetes (+27%) and Proteobacteria (+24%) (**Figure 5**). When the dataset was divided by plant species, the relative abundance of Bacteroidetes was significantly (*P<*0.05) enriched in the rhizosphere of maize (+34%), vegetables (+47%), woody plants (+38%), and other crops (+91%). The phylum of Proteobacteria was generally enhanced in the rhizosphere of all plant species except rice, such as maize (+20%), vegetables (+25%), herbaceous (+22%), and woody plants (+35%).

**Figure 6:**
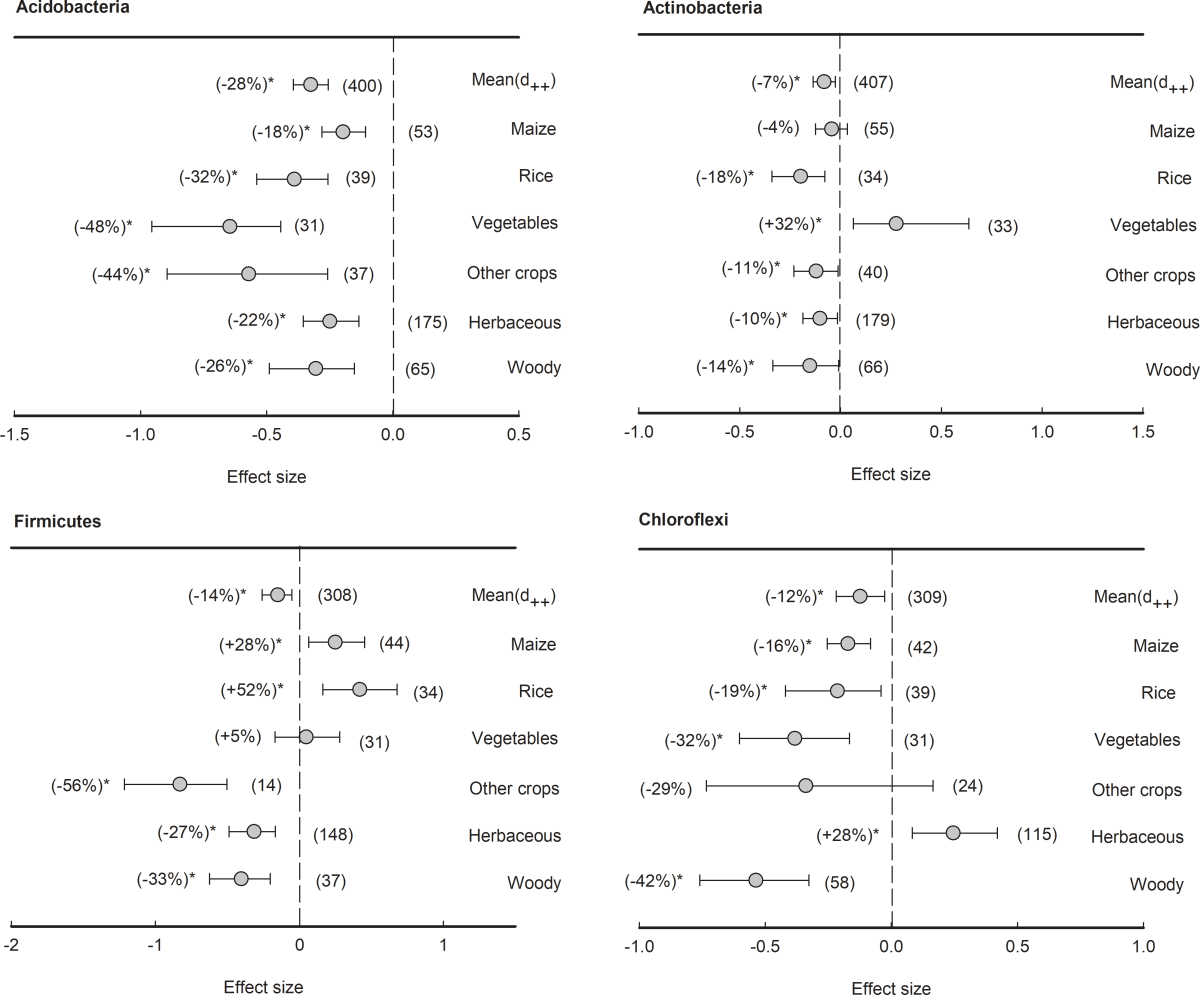
Plant rhizosphere effects on major bacterial phyla (mean proportion > 5%), i.e., Acidobacteria, Actinobacteria, Firmicutes and Chloroflexi, grouped by the plant species including maize, rice, vegetables, other crops, herbaceous and woody plants. Data is expressed as the mean effect size (d_++_) with bias-corrected 95% confidence intervals. Percentage changes for means and observation numbers for the category are given in parentheses. Asterisk indicates *P*< 0.05.

However, four bacterial phyla with average relative abundance over 5% had an overall negative effect size of plant rhizosphere across all plant species, although the reduction percentages varied, such as Acidobacteria (-28%), Actinobacteria (-7%), Firmicutes (-14%), and Chloroflexi (-12%) (**Figure 6**). Specifically, in Acidobacteria, though all plant species had negative effect size, the negative effects of rhizosphere in vegetables was the lowest (-48%), significantly (*P<*0.05) lower than those in maize (-18%) and herbaceous plants (-22%). In Actinobacteria, all plant species, with the exception of maize (-4%) and vegetables (+32%), exhibited negative effect sizes ranging from -18% to -10%. In Firmicutes, both herbaceous (-27%) and woody plants (-33%) showed negative responses to rhizosphere effects, but those in maize (28%) and rice (52%) were contrarily enriched in the rhizosphere. In Chloroflexi, apart from herbaceous plants showing a positive effect size (+28%), the plant rhizosphere effects were consistently negative across all other plant types (ranging from -42% to -16%).

**Figure 7:**
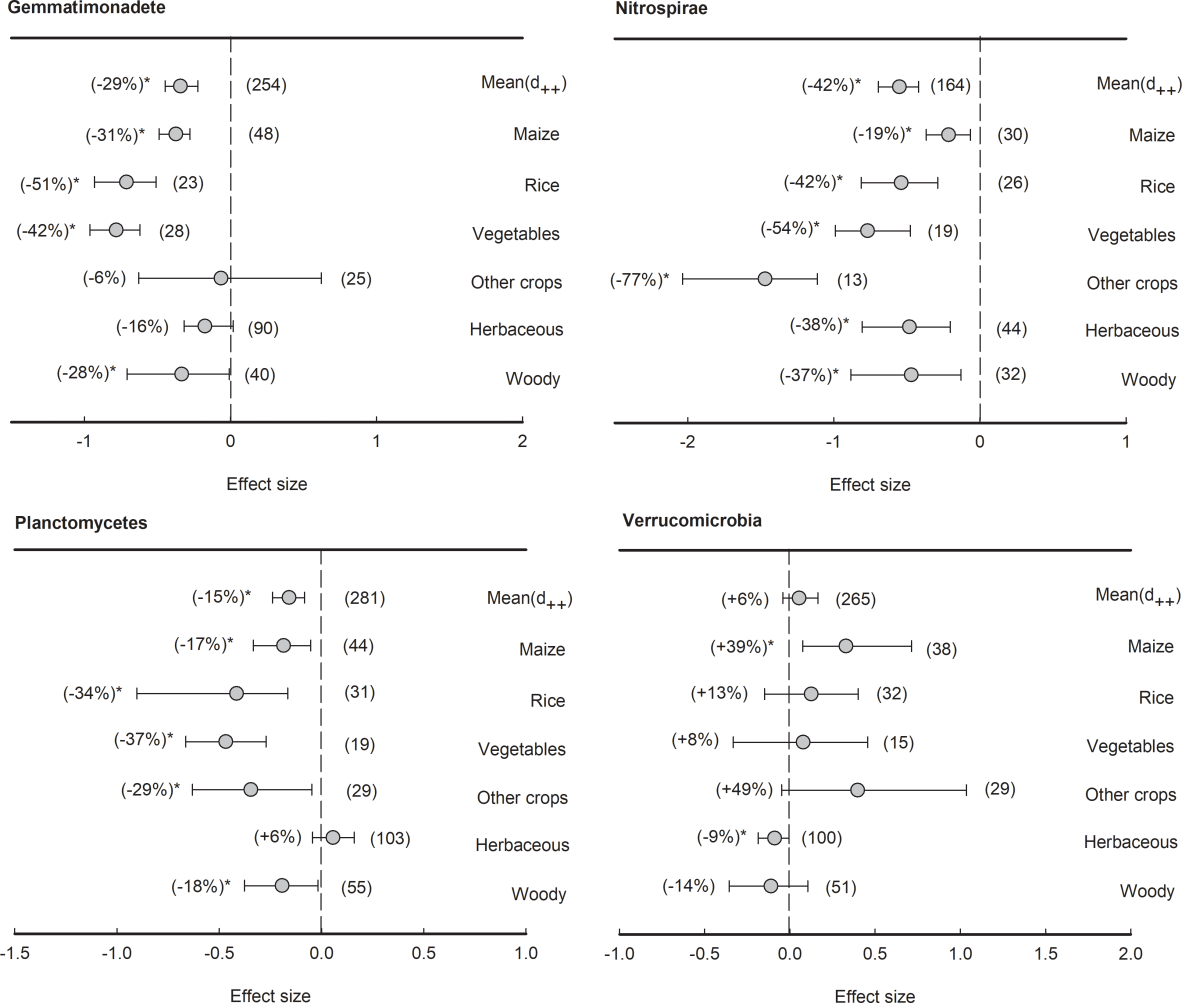
Plant rhizosphere effects on other bacterial phyla (median proportion of 1-5%), i.e., Gemmatimonadete, Nitrospirae, Planctomycetes and Verrucomicrobia, grouped by the plant species including maize, rice, vegetables, other crops, herbaceous and woody plants. Data is expressed as the mean effect size (d_++_) with bias-corrected 95% confidence intervals. Percentage changes for means and observation numbers for the category are given in parentheses. Asterisk indicates *P*< 0.05.

With an average relative abundance of less than 5%, three bacterial phyla showed negative effect size in general, including Gemmatimonadete (-29%), Nitrospirae (-42%), and Planctomycetes (-15%) (**Figure 7**). The phylum Gemmatimonadete was significantly (*P<*0.05) depleted in the rhizosphere of all plant types except herbaceous plants and other crops, such as maize (-31%), rice (-51%), vegetables (-42%), and woody plants (-28%). Furthermore, the phylum Nitrospirae was significantly (*P<*0.05) depleted in the rhizosphere of all plant species (from -77% to -19%). Similarly, in Planctomycetes, the effect sizes of rhizosphere effects were all negative across all plant types (from -37% to -17%) except herbaceous plants. Last but not least, although Verrucomicrobia showed no significant response to the rhizosphere effect on average, maize had a significantly (*P<*0.05) positive effect size (+39%), while herbaceous plants had a negative effect size in the rhizosphere (-9%).

**Figure 8:**
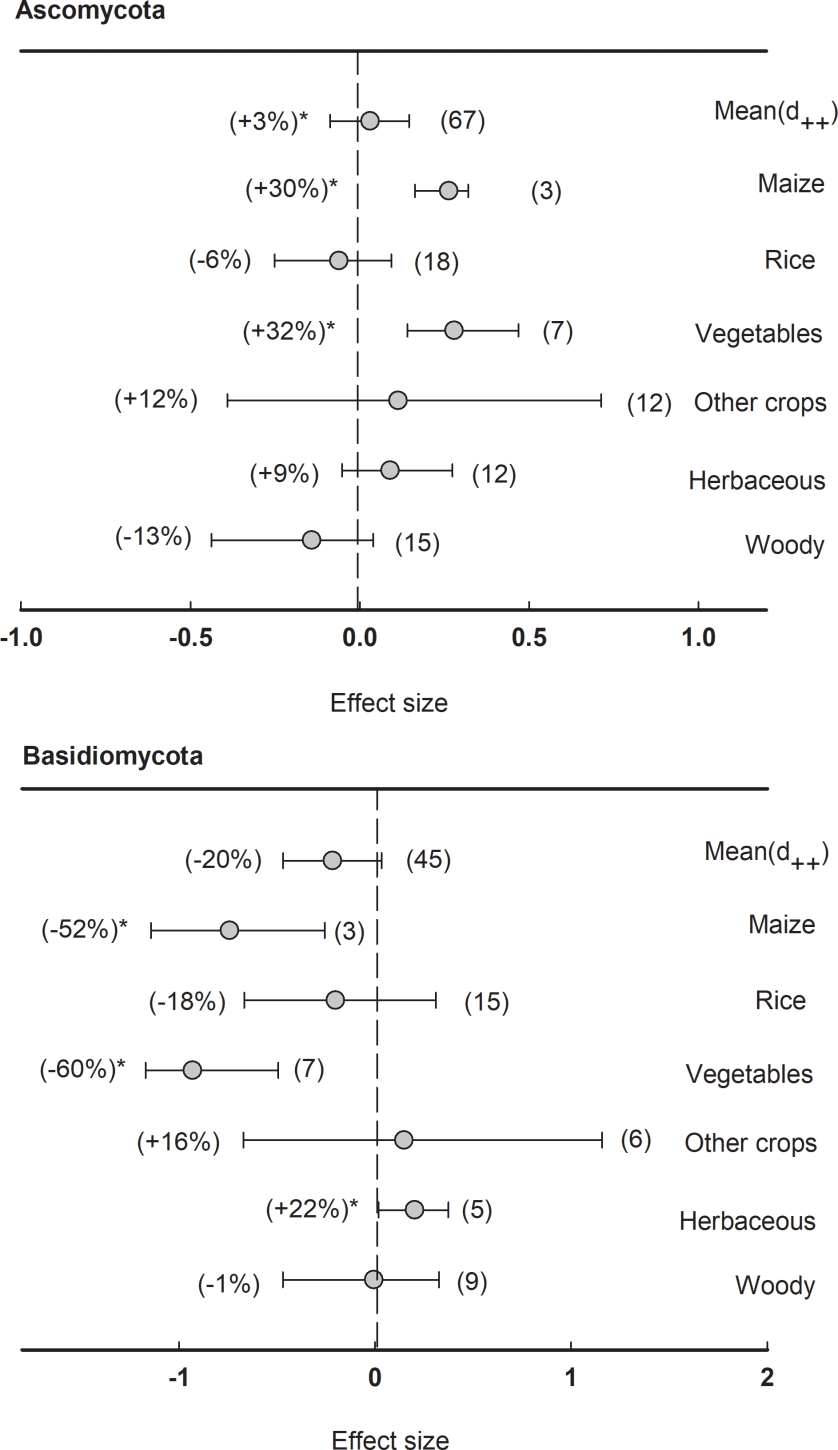
Plant rhizosphere effects on major fungal phyla (mean proportion > 5%), i.e. Ascomycota and Basidiomycota, grouped by the plant species including maize, rice, vegetables, other crops, herbaceous and woody plants. Data is expressed as the mean effect size (d_++_) with bias-corrected 95% confidence intervals. Percentage change for means and observation numbers for the category are given in parentheses. Asterisk indicates *P*< 0.05.

In fungal communities, the effect size of rhizosphere was less significant compared to bacteria. Neither Ascomycota nor Basidiomycota showed a significant (*P<*0.05) response to the rhizosphere effect (**Figure 8**). However, the phylum Ascomycota enriched in the rhizosphere of maize (+30%) and vegetables (+32%), while the phylum Basidiomycota depleted in the rhizosphere of maize (-52%) and vegetables (-60%), and enriched in herbaceous plants (+22%).

## 4 Discussion

### 4.1 Soil pH responding to rhizosphere effects in various plant species

Our meta-analysis revealed that both the effect sizes of bulk soil pH > 6 and the mean effect sizes of soil pH were significantly negative (**Figure 2**). These findings imply that most plants tend to decrease the rhizosphere soil pH compared with the bulk soil across all studies. A previous study has reported that the release of H^+^ by roots is a dominant mechanism for plants to mobilize nutrients and maintain electrochemical potential on the root surface in slightly acidic, neutral, and alkaline soils (Marschner, 2012). Under the extreme soil pH condition (too low or too high), plant roots can mitigate the constraints, such as plant roots that could alleviate Al^3+^ or Fe^3+^ toxicity in acidic pH conditions, and also Fe or Mn deficiency in alkaline pH conditions (Philippot et al., 2013; Kuzyakov and Razavi, 2019). Thus, our results that the effect sizes of rhizosphere were significantly lower than zero in soil pH > 6 further suggested that plant roots could alleviate constraints under neutral or slightly alkaline conditions. Moreover, our result showed the effect sizes of rice and vegetables were significantly lower than zero, which implies that the roots of rice and vegetables tend to acidify the rhizosphere soil more severely than other plants. This is in line with the fact that rice root exudates organic acid decreasing rhizosphere soil pH, increasing amino acid availability, and promoting ammonia release and subsequent nitrification (Dohrmann et al., 2013; Di Salvo et al., 2018). Furthermore, legumes acidify the rhizosphere soil through excess cation uptakes during N_2_ fixation and photosynthetic activity to alter cation-anion uptake ratios (Bolan et al., 1991; Rao et al., 2000; Rao et al., 2002). Similarly, it has been found that the rhizosphere soil pH of pak choi decreased by root exudates, which mainly consist of organic acids and amino acids (e.g., citric acid, ferulic acid, cinnamic acid, glutamic acid, alanine, and valine) (Kim et al., 2017; Jeon et al., 2018; Cai et al., 2019). As previous studies report most plant species tend to acidify the rhizosphere soil (Kuzyakov and Razavi, 2019), our results further provide substantial evidences to support that these pH variation may significantly contribute to the assembly of soil microbial community in plant rhizophere.

### 4.2 Distinct microbial community diversity and structure in the rhizosphere

In our analysis, PCoA of the Bray-Curtis distances showed significant clustering of bacterial communities between rhizosphere and bulk soils, with decreased community diversity (Shannon index) observed in the rhizosphere soil across different plant species (**Figures 3**, **4B**). This is consistent with previous findings that diversity decreased from the bulk soil to the roots (Poole, 2017; Semenov et al., 2019). The significant contrast between the bulk soil and rhizosphere soil is a crucial factor contributing to variations in microbiota composition (Yan et al., 2017; Ren et al., 2020). For example, Fan et al. (2017) reported that the distance decay relationship (from the root surface to bulk soil) can reflect variations in microbial community composition. In addition, it has been suggested that the decrease in diversity from the bulk soil to roots could be attributed to root “rhizosphere effect” (Bulgarelli et al., 2012; Lundberg et al., 2012; Barajas et al., 2020; Attia et al., 2022; Santoyo, 2022). Specific microorganisms are commonly selected by plant roots to colonize the rhizosphere, which can attract beneficial microorganisms to improve nutrient acquisition and combat pathogenic taxa for plants (Dennis et al., 2010; Berendsen et al., 2012; Fan et al., 2017). Generally, the rhizosphere is a highly selective environment that can select microbiome through two distinct processes (Fan et al., 2017; Fan et al., 2018). The first process involves the general recruitment of microbes to the proximity of the root, whereas the second process involves the transition of microbes from external to internal occupancy in the root (Edwards et al., 2015). Molecular signals from plants, including components of root exudates and possibly cell wall or membrane proteins (Edwards et al., 2015; Edwards et al., 2018; Kuzyakov and Razavi, 2019), are involved in the selection of microbial communities. Notably, the DNA extraction protocols for metagenomic DNA, particularly in relation to rhizosphere and soil samples, may introduce inherent biases due to variations in rhizosphere soil sampling procedures and differences arising from DNA extraction methods. Despite the presence of disparities, our study represents a comprehensive synthesis of over 100 studies, thus mitigating any potential systematic biases.

Additionally, the results of the present meta-analysis showed that rhizosphere soil microbial community structure varies depending on plant species (**Figure 4C**). This is in agreement with previous studies that the plant species and the genotypes of individual plants can exert a profound influence on the composition of their associated microbial communities in the rhizosphere (Schweitzer et al., 2008; Lau and Lennon, 2012; Bever et al., 2013). However, our meta-analysis showed the phyla Proteobacteria and Bacteroidetes are consistently enriched in the rhizosphere (**Figure 5**). This result suggests that the phyla Proteobacteria and Bacteroidetes are well-suited to the rhizosphere, which provides C-rich conditions for high metabolic activity, fast growth, and propagation (Pausch et al., 2013; Kuzyakov and Razavi, 2019). The phyla Proteobacteria and Bacteroidetes are generally considered r-strategists and copiotrophs that respond to labile C sources, and fast-growing microbiota with population opportunity fluctuations (Fierer et al., 2007; Peiffer et al., 2013). In contrast, our meta-analysis showed that the phyla Acidobacteria, Gemmatimonadete, Nitrospirae, and Planctomycetes are consistently depleted in the rhizosphere (**Figures 6**, **7**). It has been previously reported that the phyla Acidobacteria were depleted in wheat rhizosphere under the field of North China Plain (Fan et al., 2017). Moreover, a previous study reported that the phyla Acidobacteria were enriched in the bulk soil, while depleted in the rhizosphere soil under the Central European grasslands and forests (Kaiser et al., 2016). Similar results have been revealed in Mexico’s agroecosystem, German grassland, and forest soil as well (Foesel et al., 2014; Embarcadero-Jimenez et al., 2016; Dawson et al., 2017). As a result, these phyla are extremely similar in rhizosphere soil across varied plant species (Ling et al., 2022). Similarly, Planctomycetes are more abundant in the rhizosphere than in bulk soil (Fierer et al., 2007). The phyla Gemmatimonadete, Nitrospirae, and Planctomycetes have been extensively detected as K-strategists (slow-growing microbiota) and oligotrophic that are adapted to survive when resource was limited or low substrate concentrations (Fontaine et al., 2003; Bernard et al., 2007; Blackburne et al., 2007), and generally considered to be enriched in the bulk soil with less energy and nutrients compared with the rhizosphere soil. Overall, our meta-analysis implied that the phyla Proteobacteria and Bacteroidetes enriched commonly in the rhizosphere of most plant species, while Acidobacteria, Gemmatimonadete, Nitrospirae, and Planctomycetes were depleted contrarily by plant rhizosphere effects. Notably, agricultural practices might make up the plant-associated microbial communities. Tillage can lead to shifts in microbial communities as anaerobic microorganisms are exposed to oxygen (Coleman-Derr et al., 2016; Dawson et al., 2017). Reduced or no-till farming can preserve anaerobic niches and maintain a different microbial community structure. Synthetic fertilizers often provide easily accessible nutrients, which can favor certain microbial taxa, while organic fertilizers release nutrients slowly, supporting a more diverse microbial community (Yang et al., 2017). Crop rotation and the diversity of crops planted in a field can provide different root exudates and organic matter, altering the nutrient availability and microbial community. This practice promotes a more diverse and dynamic microbial community (Visioli et al., 2018; Wang et al., 2020).

Moreover, the responses of the phyla Actinobacteria, Firmicutes, Chloroflexi, and Verrucomicrobia to plant rhizosphere were highly dependent on plant species (**Figures 6**, **7**). The phyla Actinobacteria was significantly depleted in the rhizosphere of rice, herbaceous, woody, and other crops, while enriched in that of vegetables. For example, it has been found that the phylum Actinobacteria dominated the wild beet rhizosphere (Zachow et al., 2014) and lettuce rhizosphere (Blau et al., 2019). Additionally, the phylum Actinobacteria has been shown to dominate in the pak choi rhizosphere compared with the bulk soil with or without Se application (Cai et al., 2019). In addition, we showed that the phylum Chloroflexi significantly depleted in the rhizosphere of maize, rice, vegetables, and woody, while enriched in that of herbaceous plants (**Figure 6**). This is in agreement with a previous study reporting that the relative abundance of Chloroflexi increased from 2.7% to 8.0% in the rhizosphere compared to the bulk soil across 19 herbaceous plants (Dawson et al., 2017). We found that the phyla Firmicutes significantly enriched in the rhizosphere of maize and rice, while depleted in those of herbaceous, woody plants, and other crops. This is in line with previous studies observing that the phylum Firmicutes significantly increased in the rhizosphere compared with the bulk soil in maize fields at different rice growth stages (de Araujo et al., 2019; Li et al., 2019). Similarly, the abundance of Verrucomicrobia revealed opposite trends between the rhizosphere of maize and herbaceous plants (**Figure 7**), which was greatly observed in previous studies (Coleman-Derr et al., 2016; Dawson et al., 2017; Yang et al., 2017; Visioli et al., 2018; Wang et al., 2020).

Additionally, the phyla Ascomycota and Basidiomycota were the dominant fungal communities having distinct responses to rhizosphere depending on plant species (**Figure 8**), indicating that plant species differentiate their root microbiota in a species-specific manner (Berg and Smalla, 2009; Aira et al., 2010; Dawson et al., 2017). Such as, Ascomycota had a higher relative abundance in the rhizosphere of maize. In contrast, bulk soils had a higher abundance of Basidiomycota (Philippot et al., 2013). Commonly, plant species determine the structure of the rhizosphere soil microbial community as follows: firstly, the soil layer surrounding the roots promotes the growth of organotrophic microorganisms and initiates a shift in the soil microbiome through rhizodeposits and root cell wall features (Bulgarelli et al., 2013); secondly, the selection process that depends on the host genotype occurs close to the root surface (Reinhold-Hurek et al., 2015), fine-tuning the community profiles that thrive on the rhizoplane. Overall, the phyla Proteobacteria and Bacteroidetes are considered r-strategists that enriched in rhizosphere across all plant species, while Acidobacteria, Gemmatimonadete, Nitrospirae, and Planctomycetes are considered as K-strategists that depleted in rhizosphere across all plant species. Especially, Actinobacteria, Firmicutes, Chloroflexi, and Verrucomicrobia were selected in a species-specific manner from various plant species, thus revealing divergent abundance among different plants.

## 5 Conclusions

Our study demonstrates a significant distinction in the microbial community structure between the bulk and rhizosphere soils, which simultaneously vary depending on plant species. In particular, r-strategists (e.g. Proteobacteria and Bacteroidetes) enriched in the rhizosphere but K-strategists (e.g. Acidobacteria, Gemmatimonadete, Nitrospirae, and Planctomycetes) depleted in the rhizosphere. In contrast, the responses of some microbiota (e.g. Actinobacteria, Firmicutes, Chloroflexi, Verrucomicrobia, Ascomycota, and Basidiomycota) to plant rhizosphere effects were dependent on plant types through species-specific manner. This meta-analysis has revealed that plants generally exert a rhizosphere acidification effect through the release of organic acids via root exudates, which may particularly affect certain microbial species in the rhizosphere. Further investigations are needed to identify various environmental factors that influence plants and, subsequently, their influences on the rhizosphere microbiota associated with nutrient cycling and soil health.

## Data availability statement

The original contributions presented in the study are included in the article/**Supplementary Material**. Further inquiries can be directed to the corresponding authors.

## Author contributions

XF and YH contributed equally to this study. All authors contributed to the article and approved the submitted version.
