# Supplementary material for: Critical transition of soil microbial diversity and composition triggered by plant rhizosphere effects
Source: Front Plant Sci. 2023 Nov 10;14:1252821. doi: 10.3389/fpls.2023.1252821 (PMC10676204; doi:10.3389/fpls.2023.1252821)
Supplement: Supplementary file 1 [file Table_1.docx]

**Supplementary Table 1.** Basic information of studies used in this meta-analysis including literature source, experimental location, latitude (LAT) and longitude (LON), environmental properties, analyzed microbial communities, and reported alpha-diversity indices.

| Author | Plant name | Location | LAT | LON | Soil properties | Replication | Alpha-diversity | Amplification region | Sequencer imformation |
| --- | --- | --- | --- | --- | --- | --- | --- | --- | --- |
| Hasina Afroz 2019 | Rice | Valencia, Spain | 39.7 | 19.3 | Soil C, N, pH | 5 | Chao1, Shannon | V4 | illumina |
| Hanin Alzubaidy 2016 | Gray mangrove | Thuwal coast, Saudi Arabia | 22.18 | 39.5 | Soil C, N, pH | 2 | None |  | 454 GS  FLX Titanium |
| Yasir Arafat 2019 | Tea | Fuzhou, Fujian,China | 26.08 | 119.23 | Soil C, N, pH | 4 | Chao1, Observed OTUs | ITS1 | illumina |
| Khald Blau 2019 | Maize | LUFA Speyer, Speyer, Germany |  |  | Soil C, N, pH | 3 | Chao1, Shannon | V3-V4 | illumina |
| Davide Bulgarelli 2015 | Cowpea | Golm, German |  |  | Soil C, N, pH | 3 | Chao1, Shannon | V5-V6-V7 | illumina |
| Joseph E. Carrara 2018 | lettuce (Lactuca sativa L. cv. Tizian) | West Virginia, United States | 39.03 | 70.64 | Soil C, N, pH | 10 | Chao1, Observed OTUs, Shannon | ITS1F-ITS2 | illumina |
| Xue-Xue Chen 2018 | Barely | Guangzhou, China |  |  | Soil C, N, pH | 3 | Chao1, Shannon | V5+V6  ITS | illumina |
| Miaomiao Cai 2019 | Tree | Huazhong Agriculutral University, Wuhan, China | 30.28 | 114.21 | Soil C, N, pH | 4 | Chao1, Shannon |  | illumina |
| Yan Chen 2018 | Peanut | Yingtan, Jiangxi , China | 28.25 | 116.92 | Soil C, N, pH | 3 | Chao1, Shannon | V4 | illumina |
| Qing-Lin Chen 2018 | Brassica chinensis L. |  |  |  | Soil C, N, pH | 3 | Chao1, Shannon | V4-V5 | illumina |
| Lin Chen 2019 | Maize | Hailun ,China | 47.43 | 126.63 | Soil C, N, pH | 3 | Chao1, Observed OTUs, Shannon | V4 | illumina |
|  |  | Fengqiu,China | 35 | 114.4 | Soil C, N, pH | 3 | Chao1, Observed OTUs, Shannon |  |  |
|  |  | Yingtan,China | 28.25 | 116.92 | Soil C, N, pH | 3 | Chao1, Observed OTUs, Shannon |  |  |
| Yanmei Chen 2018 | H. cannabinus | Shaoguan, Guangdong, China | 24.82 | 114.34 | Soil C, N, pH | 5 | Chao1, Observed OTUs, Shannon |  | illumina |
| Jianzhong Cheng 2019 | Maize | Kaiyang,Guizhou ,China | 27 | 107.03 | Soil C, N, pH | 4 | Chao1, Observed OTUs, Shannon | V3-V4 | illumina |
| Carolina Chiellini 2019 | Beech (Fagus sylvatica L.) | Monte Zuccarello,Italy | 43.36 | 13.03 | Soil C, N, pH | 3 | Chao1, Observed OTUs, Shannon | V4 | illumina |
| Alyssa T. Cochran 2018 | Hyperaccumulator plant | Pine Ridge,Fort Collins, CO |  |  | Soil C, N, pH | 6 | Chao1, Shannon |  | illumina |
| Devin Coleman-Derr 2016 | Agave | Guanajuato,Mexico |  |  | Soil C, N, pH | 3 | Chao1, Shannon | ITS2 | illumina |
|  |  | California, USA |  |  | Soil C, N, pH | 3 | Chao1, Shannon |  |  |
| Catarina Cúcio 2016 | Seagrass | Faro,Portugal | 36.59 | 7.49 | Soil C, N, pH | 5 | Chao1, Shannon | V3-V4 | illumina |
|  |  | Roscoff,France | 48.43 | 3.58 | Soil C, N, pH | 5 | Chao1, Shannon |  |  |
| Er-Ping Cui 2018 | Maize | Xinxiang, Henan Provinc | 35.32 | 113.88 | Soil C, N, pH | 3 | Chao1, Shannon | V3-V4 | illumina |
| Wayne Dawson 2017 | Agrostis capillaris/Brachypodium sylvaticum/Dactylis glomerata/Deschampsia cespitosa/Phleum pratense/Centaurea jacea/Cirsium oleraceum/Jacobaea vulgaris/Pulicaria dysenterica/Taraxacum officinale/Rumex maritimus/Rumex obtusifolius/Silena alba/Silene vulgaris/Plantago lanceolata/Plantago major/Verbascum thapsus/Salvia pratensis/Lotus corniculatus | Switzerland and southern Germany |  |  | Soil C, N, pH | 5 | Chao1, Shannon | V1–V3 | illumina |
| Salvador Embarcadero-Jiménez 2016 | P. olereacea L. | Xochimilco,San Gregorio, Atlapulco, Mexico | 19.23 | 99.07 | Soil C, N, pH | 3 | Chao1, Shannon | V1-V3 | illumina |
| Eunice Essela 2019 | Pea | Dingxi, Gansu Province, China | 35.47 | 104.73 | Soil C, N, pH | 3 | Chao1, Shannon | V3-V4  ITS3-ITS4 | illumina |
| Jennifer Estendorfer1 2017 | Dactylis glomerata | Southwest of German |  |  | Soil C, N, pH | 3 | Chao1, Shannon | V2-V4 | illumina |
| Miaochun Fan 2018 | Robinia pseudoacacia | Mianxian, Shaanxi,China | 33.12 | 106.8 | Soil C, N, pH | 10 | Chao1, Shannon | V4-V5 | illumina |
| Kunkun Fan 2017 | Wheat | North China Plain | 32 | 110 | Soil C, N, pH | 5 | Chao1, Shannon | V4-V5 | illumina |
| Citlali Fonseca-García 2016 | Myrtillocactus geometrizans | EI Magueyal, Guanajuato, Mexico | 21.08 | 100.17 | Soil C, N, pH | 3 | Chao1, Shannon | V4  ITS2 | illumina |
|  | Opuntia obusta | San Felie, Guanajuato, Mexico | 21.39 | 100.2 | Soil C, N, pH | 3 | Chao1, Shannon |  |  |
| Jiangli Gao 2019 | Caragana microphylla | Northern China Desert Region |  |  | Soil C, N, pH | 3 | Chao1, Shannon | V4-V5 | illumina |
| Fabienne Gremion 2003 | Thlaspi caerulescens | Ticino, Switzerland |  |  | Soil C, N, pH | 3 | Chao1, Shannon | 16S(27F-1492R) | illumina |
| Dongchu Guo 2019 | L. Lucidum/B. papyrifera | Lengshuijiang, Hunan,China |  |  | Soil C, N, pH | 3 | Chao1, Shannon | V3-V4 | illumina |
| HE Huaidong 2017 | Oryza sativa L.(rice,cultivar Tianyou 122 ) | Liantang Village, Qingyuan,Guangdong, China | 23.87 | 113.6 | Soil C, N, pH | 3 | Chao1, Shannon | V3–V4 | illumina |
|  | Oryza sativa L.(rice,cultivar Tianyou 122 ) | Fankou , Shaoguan, Guangdong, China | 25.12 | 113.65 | Soil C, N, pH | 3 | Chao1, Shannon | V3–V4 | illumina |
| Yanhui He 2019 | Pepper (Capsicum annuum L,) | Shihezi University,Xinjiang, China | 44.38 | 85.68 | Soil C, N, pH | 3 | Chao1, Shannon | V3–V4 | illumina |
| Linnea K. Honekert 2019 | Buffalo grass | Dewey-Humboldt, AZ |  |  | Soil C, N, pH | 3 | Chao1, Shannon | V4 | illumina |
| Jinyu Hou 2019 | Sedum plumbizincicola | Renhua,Guangdong,China | 25.1 | 113.66 | Soil C, N, pH | 10 | Chao1, Shannon | V4–V5 | illumina |
| Dandi Hou 2018 | Rice (Oryza sativa L.) | Shaoxing, Zhejiang, China |  |  | Soil C, N, pH | 3 | Chao1, Shannon | V3-V4 | illumina |
| Jinxiang Hu 2016 | Pepino (Solanum muricatum) | Shilin Yi Autonomous County,Yunnan,China | 24.8385 | 103.625683 | Soil C, N, pH | 3 | Chao1, Shannon | V3-V4 | illumina |
| Cai Hui 2018 | Whaet | Jintan, Jiangsu, China | 31.39 | 119.28 | Soil C, N, pH | 3 | Chao1, Shannon | V3-V4  ITS5-ITS2 | illumina |
| Xiao-Tao Jiang 2013 | Mangrove | Hong Kong, SAR, China | 22.82 | 114.07 | Soil C, N, pH | 3 | Chao1, Shannon | V6 | illumina |
| Yuji Jiang 2017 | Rabbiteye Blueberry(Vaccinium ashei Reade) /Blackberry (Rubus spp.) | Lishui, Nanjing, China | 32.08 | 119.03 | Soil C, N, pH | 3 | Chao1 | V4-V5 | illumina |
| Myung-Suk Kang 2019 | Sedum takesimense Nakai/Campanula takesimana Nakai | Ulleungdo/Dokdo, Republic of Korea |  |  | Soil C, N, pH | 2 | Chao1, Shannon | V3-V4 | illumina |
| Akitomo Kawasaki 2016 | Brachypodium distachyon | Canberra,ACT, Australia | 35.33 | 149.1 | Soil C, N, pH | 5 | Chao1, Shannon | V5-V9  ITS1-ITS4 | illumina |
| Vinod Kumar 2018 | Barely/Alfala |  |  |  | Soil C, N, pH | 3 | Chao1 | V4 | illumina |
| Lorena M. Lagos 2016 | Ryegrass (Lolium perenne var. Nui) | Los Ríos region ,Chile | 40.20 | 72.35 | Soil C, N, pH | 3 | Shannon | V7-V9 | illumina |
| Shin Ae Lee 2019 | Tomato | South Korea |  |  | Soil C, N, pH | 3 | Chao1, Shannon | V4-v5  ITS3-ITS4 | illumina |
| Peng Li 2018 | Rice | Shanghai Academy of Agricultural Sciences |  |  | Soil C, N, pH | 3 | Chao1, Shannon | V8-V9 | illumina |
| Zhigang Li 2016 | Black pepper(Piper nigrum L. cv. Reyin No. 1) | Wanning City, Hainan Province, China |  |  | Soil C, N, pH | 3 | Chao1, Shannon | V4  ITS1-ITS2 | illumina |
| Hu Li 2019 | Rice | Taoyuan,Hunan,China | 28.92 | 111.45 | Soil C, N, pH | 3 | Chao1, Shannon | V4-V5 | illumina |
| Junjie Liu 2017 | Soybean | Heilongjiang, Chian | 50.25 | 127.46 | Soil C, N, pH | 12 | Chao1, Shannon | V3 | illumina |
| Jinliang Liu 2018 | Platycladus orientalis | Shananxi, China | 35.59 | 109.25 | Soil C, N, pH | 3 | Chao1, Shannon | V3-V4  ITS1-ITS2 | illumina |
| Yuan Liu 2019 | Miscanthus × giganteus | Germany | 48.75 | 8.92 | Soil C, N, pH | 4 | Chao1, Shannon | V4 | illumina |
| Junjie Liu 2018 | Soybean | Heilongjiang, Chian | 50.25 | 127.46 | Soil C, N, pH | 10 | Chao1, Shannon | ITS1-ITS2 | illumina |
| Lucas D. Lopes 2016 | Sugarcane(SP-3250) | Piracicaba,Brazil |  |  | Soil C, N, pH | 6 | Chao1, Shannon | V3-V4 | illumina |
| Jipeng Luo 2017 | Sedum alfredii | Jiaxing, ZheJjiang,China | 30.4 | 120.81 | Soil C, N, pH | 4 | Chao1, Shannon | V3-V4 | illumina |
| Jipeng Luo 2019 | Sedum alfredii | Hangzhou, Zhejiang, China |  |  | Soil C, N, pH | 4 | Chao1, Shannon | V3–V4,V4-V5 | illumina |
|  | Sedum alfredii | Quzhou, ZheJiang, China |  |  | Soil C, N, pH | 4 | Chao1, Shannon |  |  |
| Newton Z. Lupwayi 2018 | Black medic(cultivar George)  Flax (cv CDC Bethune) x Black medic(cultivar George)  Oats (cultivar Pinnacle) x Black medic(cultivar George)  Winter wheat (cultivar Unity) x Black medic(cultivar George) | Indian Head, Saskatchewan, Canada | 50.58 | 103.78 | Soil C, N, pH | 3 | Chao1, Shannon | 28F-219R | illumina |
| Ming Ma 2019 | Rice | Yangshikeng, Chongqing, China | 108.5 | 28.34 | Soil C, N, pH | 3 | Chao1, Shannon | V3-V4、V4-V5 | illumina |
| Francesca Mapelli 2018 | S. oppositifolia | Ny Ålesund, Svalbard | 78.88 |  | Soil C, N, pH | 3 | Chao1, Shannon | V4-V5 | illumina |
| Maria J. Mosqueira 2019 | Date palm (Phoenix dactylifera cultivar Deglet Nour) | Tunisia | 36.34 | 1.01 | Soil C, N, pH | 5 | Chao1, Shannon | V3-V4 | illumina |
| Min-Jung Kwak 2018 | Tomato(cultivar Korean cabbage)  Tomato(cultivar Hawaii 7996)  Tomato(cultivar Moneymaker) |  |  |  | Soil C, N, pH | 3 | Chao1, Shannon | V1-V3 | illumina |
| Erin E. Nuccio 2016 | Wild oat (Avena spp.) | Santa Barbara, California,USA | 34.7 | 120.03 | Soil C, N, pH | 4 | Chao1, Shannon | V4 | illumina |
|  |  | Yuba, California, USA | 39.23 | 121.3 | Soil C, N, pH | 4 | Chao1, Shannon |  |  |
|  |  | Mendocino, California, USA | 38.98 | 123.06 | Soil C, N, pH | 4 | Chao1, Shannon |  |  |
| Tanzelle Oberholster 2017 | Sorghum (cultivar K2)  Sunflower (cultivar PAN 2057) | South Africa |  |  | Soil C, N, pH | 5 | Chao1, Shannon | V4-V5 | illumina |
| Javier Pascual 2017 | Thymus zygis L. | Sierra Nevada National Park, Granada, Spain |  |  | Soil C, N, pH | 3 | Chao1, Shannon | ITS86F-ITSR2 | illumina |
| Shamina I. Pathan 2015 | Maize | Cesa, Tuscany, Italy |  |  | Soil C, N, pH | 5 | Chao1, Shannon |  | illumina |
| Hoang Nam Pham 2017 | 1. Vittata   D. linearis | Ha Thuong Commune,Dai Tu district, Thai Nguyen, Vietnam | 21.86 | 105.80 | Soil C, N, pH | 3 | Chao1 |  | illumina |
|  | P. vittata | Hoang Quoc Viet Street, Cau Giay district, Hanoi, Vietnam | 21.86 | 105.80 | Soil C, N, pH | 3 | Chao1 |  |  |
| Qinghua Qiao 2019 | Cotton:G. hirsutum(cultivar TM-7, upland cotton), G.barbadense(cultivar Hai 7124, island cotton with higher disease resistance) | Shandong Province, China | 36.81 | 115.71 | Soil C, N, pH | 3 | Chao1, Shannon | ITS1 | illumina |
| Qinghua Qiao 2017 | Cotton:G. hirsutum(cultivar TM-7, upland cotton), G.barbadense(cultivar Hai 7124, island cotton with higher disease resistance) | Shandong Province, China | 36.81 | 115.71 | Soil C, N, pH | 3 | Chao1, Shannon | V4 | illumina |
| Hua Qin 2014 | Sudangrass (Sorghum sudanese) | Zhejiang Province, China |  |  | Soil C, N, pH | 3 | Chao1, Shannon | V4-V5 | illumina |
| Chanz Robbins 2017 | A. thaliana | Julius Kuhn Experimental Station | 51.49 | 11.99 | Soil C, N, pH | 3 | Chao1, Shannon | V5-V7  ITS1F-ITS2 | illumina |
| Robertson-Albertyn 2017 | Barely | Kingoodie, Scotland, United Kingdom | 56.45 | 3.07 | Soil C, N, pH | 5 | Chao1, Shannon | V4 | illumina |
|  |  | Tayport, Scotland, United Kingdom | 56.42 | 2.88 | Soil C, N, pH | 5 | Chao1, Shannon |  |  |
| Xavier Rojas 2016 | Tall fescue | Kentucky, USA |  |  | Soil C, N, pH | 7 | Chao1, Shannon | V4  ITS1F-ITS2 | illumina |
| Tianyun Shao 2019 | Jerusalem artichoke | Dafeng, Jiangsu, China | 32.92 | 120.81 | Soil C, N, pH | 3 | Chao1, Shannon | V3-V4 | illumina |
| Zongzhuan Shen 2019 | Banana | Hainan province, China | 18.38 | 108.47 | Soil C, N, pH | 3 | Chao1, Shannon | V4 | illumina |
| Matthew Shenton 2016 | Rice | National Institute of Genetics, Japan |  |  | Soil C, N, pH | 3 | Chao1, Shannon | V4 | illumina |
| Shengjing Shi 2015 | Avena fatua | Little Buck watershed at the University of California Hopland Research and Extension Center |  |  | Soil C, N, pH | 16 | Chao1, Shannon | V4 | illumina |
| Jugpreet Singh 2019 | Apple(HoneyCrisp) | Newark, Wayne County, New York, USA | 43.12 | 77.09 | Soil C, N, pH | 6 | Chao1, Shannon | V3-V4  ITS1F-ITS4R | illumina |
| Jianqiang Su 2016 | Spartina alterniflora | Jiulong River Estuary Mangrove Nature Reserve, China | 24.24 | 117.55 | Soil C, N, pH | 3 | Chao1, Shannon | V3 | illumina |
| Xiaoyan Sun 2018 | P. purpereum  T.angustifolia  A. cremastogyne | Sizhou, Dexing, Jiangxi, China | 29.06 | 117.73 | Soil C, N, pH | 3 | Chao1, Shannon | V3-V4 | illumina |
| Jian Sun 2016 | Pingyitiancha | Bohai Bay area,Beijing,China |  |  | Soil C, N, pH | 3 | Chao1, Shannon | ITS1F-ITS2 | illumina |
| Márton Szoboszlay 2019 | Wheat(cultivar Opata) | West of the city of La Paz, Baja California, | 24.07 | 110.26 | Soil C, N, pH | 3 | Chao1, Shannon | V4 | illumina |
| Yong Tan 2016 | Panax notoginseng | Wenshan, Yunnan, China | 104.2 | 23.63 | Soil C, N, pH | 3 | Chao1, Shannon | ITS1F-ITS2 | illumina |
| François Thomas 2016 | Ryegrass |  |  |  | Soil C, N, pH | 3 | Chao1, Shannon | V3-V4 | illumina |
| Marika Truu 2017 | Betula pendula Roth(silver birch) | Rõka village , Järvselja Experimental Forest District, Estonia | 58.41 | 27.3 | Soil C, N, pH | 4 | Chao1, ACE | V6 | illumina |
| Abid Ullah 2019 | Cotton plants (Gossypium hirsutum cv. Jin668) u |  |  |  | Soil C, N, pH | 3 | Chao1, ACE | V3-V4 | illumina |
| Stéphane Uroz 2010 | Oak forest | Breuil-Chenue, France | 47.3 | 1.083 | Soil C, N, pH | 1 | Chao1, Shannon | V5-V6 |  |
| Giovanna Visioli 2018 | Maize (hybrid SY-Hydro Syngenta, Basel, Switzerland) | Chivasso, Turin, Italy | 45.21 | 7.92 | Soil C, N, pH | 4 | Chao1, Shannon | V3 | illumina |
| Qingfeng Wang 2018 | Maize (a long-term (36-year) fertilizer experiment) | Harbin, Heilongjiang, China | 45.67 | 126.58 | Soil C, N, pH | 3 | Chao1, Shannon | V3-V4 | illumina |
| Peng Wang 2019 | Maize hybird(released from 1995 to 2012)  Maize hybird(released from 1958 to 1983) | Brule, Nebraska, USA | 41.09 | 101.89 | Soil C, N, pH | 4 | Chao1, Shannon | V4 | illumina |
| Cassandra J. Wattenburger 2019 | Maize | Boone, IA | 42.01 | 93.78 | Soil C, N, pH | 9 | Chao1, Shannon | V4，V11 | illumina |
| Xiaomeng Wei 2019 | Rice | Hunan Province, China | 28.33 | 113.19 | Soil C, N, pH | 3 | Chao1, Shannon | ALPS-F730-  ALPS-R1101 | illumina |
| Max E. Winston 2014 | Cannabis | Vista, California, USA |  |  | Soil C, N, pH | 3 | Chao1, Shannon | V4 | illumina |
| Kosuke Yamamoto 2018 | Glaux maritima  Salicornia europaea | Hokkaido, Japan | 44.04 | 144.19 | Soil C, N, pH | 4 | Chao1, Shannon | V3-V4 | illumina |
| Yi Yang 2017 | Maize (Zea mays L.) | Ministry of Environmental Protection, China |  |  | Soil C, N, pH | 3 | Chao1, Shannon | V3-V4 | illumina |
| Etienne Yergeau 2014 | Willow(Salix purpurea cultivar Fish Creek) | Varennes, QC, Canada | 45.69 | 73.43 | Soil C, N, pH | 6 | Chao1, Shannon | V3 | illumina |
| Svetlana N. Yurgel 2018 | Wild Blueberry | Nova, Scotia, Canada |  |  | Soil C, N, pH | 5 | Chao1, Observed OTUs, Shannon | V4，V6-V8 | illumina |
| Christin Zachow 2014 | Coastal drift line soil  Wild beet plants  Commercial sugar beet plant in coastal drift line soil  Commercial sugar beet plant | Mediterranean Sea coast, Slovenia | 45.59 | 13.71 | Soil C, N, pH | 3 | Chao1, Shannon | V4-V5 | illumina |
| MeijuanZeng 2018 | Baphicacanthus cusia(NeeS) | Shufeng domination Farm, Fujian, China | 25.41 | 118.65 | Soil C, N, pH | 3 | Chao1, Shannon | V4 | illumina |
| Xiaoxia Zhang 2017 | Rice | Qiyang, Hunan, China | 26.76 | 111.87 | Soil C, N, pH | 3 | Chao1, Shannon | V4 | illumina |
| Baogang Zhang 2018 | Soybean | China | 19 | 81 | Soil C, N, pH | 3 | Chao1, Shannon | V4 | illumina |
| Miaomiao Cai 2019 | Pak choi (Brassica campestris L. ssp. Chinensis Makino) | Huazhong Agriculutral University, Wuhan, China | 113.2 | 114.41 | Soil C, N, pH | 4 | Chao1, Shannon | V3-V4 | illumina |
| Hao Zheng 2018 | Seashore mallow (Kosteletzkya virginica) | Yellow River Delta, China | 37.42 | 118.67 | Soil C, N, pH | 3 | Chao1, Shannon | V4-V5 | illumina |
| Ping Zhu 2018 | Mangrove | Golden Bay Mangrove Reserve, Guangxi, China | 21.40 | 109.15 | Soil C, N, pH | 5 | Chao1, Shannon | V4 | illumina |
| Wenxu Zhu 2016 | Poplar (Populus spp.) | Beijing, China |  |  | Soil C, N, pH | 3 | Chao1, Shannon | V4 | illumina |

^#^Supplementary references: List of 101 publications used in this meta-analysis

1. Afroz, H., Su, S., Carey, M., Meharg, A.A., and Meharg, C. (2019). Inhibition of Microbial Methylation via arsM in the Rhizosphere: Arsenic Speciation in the Soil to Plant Continuum. *Environmental Science and Technology* 53(7), 3451-3463.

2. Alzubaidy, H., Essack, M., Malas, T.B., Bokhari, A., Motwalli, O., Kamanu, F.K., et al. (2016). Rhizosphere microbiome metagenomics of gray mangroves (Avicennia marina) in the Red Sea. *Gene* 576(2 Pt 1), 626-636.

3. Arafat, Y., Tayyab, M., Khan, M.U., Chen, T., Amjad, H., Awais, S., et al. (2019). Long-Term Monoculture Negatively Regulates Fungal Community Composition and Abundance of Tea Orchards. *Agronomy* 9(8), 466.

4. Blau, K., Jacquiod, S., Sorensen, S.J., Su, J.Q., Zhu, Y.G., Smalla, K., et al. (2019). Manure and Doxycycline Affect the Bacterial Community and Its Resistome in Lettuce Rhizosphere and Bulk Soil. *Frontiers in Microbiology* 10(1), 725.

5. Bulgarelli, D., Garrido-Oter, R., Munch, P.C., Weiman, A., Droge, J., Pan, Y., et al. (2015). Structure and function of the bacterial root microbiota in wild and domesticated barley. *Cell Host and Microbe* 17(3), 392-403.

6. Cai, M., Hu, C., Wang, X., Zhao, Y., Jia, W., Sun, X., et al. (2019). Selenium induces changes of rhizosphere bacterial characteristics and enzyme activities affecting chromium/selenium uptake by pak choi (Brassica campestris L. ssp. Chinensis Makino) in chromium contaminated soil. *Environment Polluttion* 249, 716-727.

7. Carrara, J.E., Walter, C.A., Hawkins, J.S., Peterjohn, W.T., Averill, C., and Brzostek, E.R. (2018). Interactions among plants, bacteria, and fungi reduce extracellular enzyme activities under long-term N fertilization. *Global Change Biology* 24(6), 2721-2734.

8. Chen, L., Xin, X., Zhang, J., Redmile-Gordon, M., Nie, G., and Wang, Q. (2019). Soil Characteristics Overwhelm Cultivar Effects on the Structure and Assembly of Root-Associated Microbiomes of Modern Maize. *Pedosphere* 29(3), 360-373.

9. Chen, Q.-L., Fan, X.-T., Zhu, D., An, X.-L., Su, J.-Q., and Cui, L. (2018a). Effect of biochar amendment on the alleviation of antibiotic resistance in soil and phyllosphere of Brassica chinensis L. *Soil Biology and Biochemistry* 119, 74-82.

10. Chen, X.X., Wu, Y., Huang, X.P., Lu, H., Zhao, H.M., Mo, C.H., et al. (2018b). Variations in microbial community and di-(2-ethylhexyl) phthalate (DEHP) dissipation in different rhizospheric compartments between low- and high-DEHP accumulating cultivars of rice (Oryza sativa L.). *Ecotoxicology and Environmental Safety* 163, 567-576.

11. Chen, Y., Sun, R., Sun, T., Liang, Y., Jiang, Y., and Sun, B. (2018d). Organic amendments shift the phosphorus-correlated microbial co-occurrence pattern in the peanut rhizosphere network during long-term fertilization regimes. *Applied Soil Ecology* 124, 229-239.

12. Cheng, J., Lee, X., Tang, Y., and Zhang, Q. (2019). Long-term effects of biochar amendment on rhizosphere and bulk soil microbial communities in a karst region, southwest China. *Applied Soil Ecology* 140, 126-134.

13. Chiellini, C., Cardelli, V., De Feudis, M., Corti, G., Cocco, S., Agnelli, A., et al. (2019). Exploring the links between bacterial communities and magnetic susceptibility in bulk soil and rhizosphere of beech (Fagus sylvatica L.). *Applied Soil Ecology* 138, 69-79.

14. Cochran, A.T., Bauer, J., Metcalf, J.L., Lovecka, P., Sura de Jong, M., Warris, S., et al. (2018). Plant Selenium Hyperaccumulation Affects Rhizosphere: Enhanced Species Richness and Altered Species Composition. *Phytobiomes Journal* 2(2), 82-91.

15. Coleman-Derr, D., Desgarennes, D., Fonseca-Garcia, C., Gross, S., Clingenpeel, S., Woyke, T., et al. (2016). Plant compartment and biogeography affect microbiome composition in cultivated and native Agave species. *New Phytologist* 209(2), 798-811.

16. Cucio, C., Engelen, A.H., Costa, R., and Muyzer, G. (2016). Rhizosphere Microbiomes of European + Seagrasses Are Selected by the Plant, But Are Not Species Specific. *Frontiers in Microbiology* 7, 440.

17. Cui, E.P., Gao, F., Liu, Y., Fan, X.Y., Li, Z.Y., Du, Z.J., et al. (2018). Amendment soil with biochar to control antibiotic resistance genes under unconventional water resources irrigation: Proceed with caution. *Environment Polluttion* 240, 475-484.

18. Dawson, W., Hor, J., Egert, M., van Kleunen, M., and Pester, M. (2017). A Small Number of Low-abundance Bacteria Dominate Plant Species-specific Responses during Rhizosphere Colonization. *Frontiers in Microbiology* 8, 975.

19. Embarcadero-Jiménez, S., Rivera-Orduña, F.N., and Wang, E.T. (2016). Bacterial communities estimated by pyrosequencing in the soils of chinampa, a traditional sustainable agro-ecosystem in Mexico. *Journal of Soils and Sediments* 16(3), 1001-1011.

20. Essel, E., Xie, J., Deng, C., Peng, Z., Wang, J., Shen, J., et al. (2019). Bacterial and fungal diversity in rhizosphere and bulk soil under different long-term tillage and cereal/legume rotation. *Soil and Tillage Research* 194, 104302.

21. Estendorfer, J., Stempfhuber, B., Haury, P., Vestergaard, G., Rillig, M.C., Joshi, J., et al. (2017). The Influence of Land Use Intensity on the Plant-Associated Microbiome of Dactylis glomerata L. *Frontiers in Plant Science* 8, 930.

22. Fan, K., Cardona, C., Li, Y., Shi, Y., Xiang, X., Shen, C., et al. (2017). Rhizosphere-associated bacterial network structure and spatial distribution differ significantly from bulk soil in wheat crop fields. *Soil Biology and Biochemistry* 113, 275-284.

23. Fan, K., Weisenhorn, P., Gilbert, J.A., Shi, Y., Bai, Y., and Chu, H. (2018a). Soil pH correlates with the co-occurrence and assemblage process of diazotrophic communities in rhizosphere and bulk soils of wheat fields. *Soil Biology and Biochemistry* 121, 185-192.

24. Fan, M., Xiao, X., Guo, Y., Zhang, J., Wang, E., Chen, W., et al. (2018b). Enhanced phytoremdiation of Robinia pseudoacacia in heavy metal-contaminated soils with rhizobia and the associated bacterial community structure and function. *Chemosphere* 197, 729-740.

25. Fonseca-Garcia, C., Coleman-Derr, D., Garrido, E., Visel, A., Tringe, S.G., and Partida-Martinez, L.P. (2016). The Cacti Microbiome: Interplay between Habitat-Filtering and Host-Specificity. *Frontiers in Microbiology* 7, 150.

26. Gao, J., Luo, Y., Wei, Y., Huang, Y., Zhang, H., He, W., et al. (2019). Effect of aridity and dune type on rhizosphere soil bacterial communities of Caragana microphylla in desert regions of northern China. *PLoS One* 14(10), e0224195.

27. Gremion, F., Chatzinotas, A., and Harms, H. (2003). Comparative 16S rDNA and 16S rRNA sequence analysis indicates that Actinobacteria might be a dominant part of the metabolically active bacteria in heavy metal-contaminated bulk and rhizosphere soil. *Environmental Microbiology* 5(10), 896-907.

28. Guo, D., Fan, Z., Lu, S., Ma, Y., Nie, X., Tong, F., et al. (2019). Changes in rhizosphere bacterial communities during remediation of heavy metal-accumulating plants around the Xikuangshan mine in southern China. *Scientific reports* 9(1), 1947.

29. He, H., Li, W., Yu, R., and Ye, Z. (2017). Illumina-Based Analysis of Bulk and Rhizosphere Soil Bacterial Communities in Paddy Fields Under Mixed Heavy Metal Contamination. *Pedosphere* 27(3), 569-578.

30. He, Y., Wu, Z., Wang, W., Liu, X., and Ye, B.-C. (2019). Bacterial community and phosphorus species changes in pepper rhizosphere soils after Pseudomonas putida Rs-198 inoculation. *Rhizosphere* 11, 100164.

31. Honeker, L.K., Gullo, C.F., Neilson, J.W., Chorover, J., and Maier, R.M. (2019). Effect of Re-acidification on Buffalo Grass Rhizosphere and Bulk Microbial Communities During Phytostabilization of Metalliferous Mine Tailings. *Frontiers in Microbiology* 10, 1209.

32. Hou, D., Wang, R., Gao, X., Wang, K., Lin, Z., Ge, J., et al. (2018). Cultivar-specific response of bacterial community to cadmium contamination in the rhizosphere of rice (Oryza sativa L.). *Environment Polluttion* 241, 63-73.

33. Hou, J., Liu, W., Wu, L., Ge, Y., Hu, P., Li, Z., et al. (2019). Rhodococcus sp. NSX2 modulates the phytoremediation efficiency of a trace metal-contaminated soil by reshaping the rhizosphere microbiome. *Applied Soil Ecology* 133, 62-69.

34. Hu, J., Yang, H., Long, X., Liu, Z., and Rengel, Z. (2016). Pepino (Solanum muricatum) planting increased diversity and abundance of bacterial communities in karst area. *Scientific reports* 6(1), 21938.

35. Hui, C., Sun, P., Guo, X., Jiang, H., Zhao, Y., and Xu, L. (2018). Shifts in microbial community structure and soil nitrogen mineralization following short-term soil amendment with the ammonifier Bacillus amyloliquefaciens DT.  *International Biodeterioration and Biodegradation* 132, 40-48.

36. Jiang, X.T., Peng, X., Deng, G.H., Sheng, H.F., Wang, Y., Zhou, H.W., et al. (2013). Illumina sequencing of 16S rRNA tag revealed spatial variations of bacterial communities in a mangrove wetland. *Microbial ecology* 66(1), 96-104.

37. Jiang, Y., Li, S., Li, R., Zhang, J., Liu, Y., Lv, L., et al. (2017). Plant cultivars imprint the rhizosphere bacterial community composition and association networks. *Soil Biology and Biochemistry* 109, 145-155.

38. Kang, M.S., Hur, M., and Park, S.J. (2019). Rhizocompartments and environmental factors affect microbial composition and variation in native plants. *Journal of Microbiology* 57(7), 550-561.

39. Kawasaki, A., Donn, S., Ryan, P.R., Mathesius, U., Devilla, R., Jones, A., et al. (2016). Microbiome and Exudates of the Root and Rhizosphere of Brachypodium distachyon, a Model for Wheat. *PLoS One* 11(10), e0164533.

40. Kumar, V., AlMomin, S., Al-Aqeel, H., Al-Salameen, F., Nair, S., and Shajan, A. (2018). Metagenomic analysis of rhizosphere microflora of oil-contaminated soil planted with barley and alfalfa. *PLoS One* 13(8), e0202127.

41. Kwak, M.J., Kong, H.G., Choi, K., Kwon, S.K., Song, J.Y., Lee, J., et al. (2018). Rhizosphere microbiome structure alters to enable wilt resistance in tomato. *Nature biotechnology* *36*(11), 1100-1109.

42. Lagos, L.M., Acuña, J.J., Maruyama, F., Ogram, A., de la Luz Mora, M., and Jorquera, M.A. (2016). Effect of phosphorus addition on total and alkaline phosphomonoesterase-harboring bacterial populations in ryegrass rhizosphere microsites. *Biology and Fertility of Soils* 52(7), 1007-1019.

43. Lee, S.A., Kim, Y., Kim, J.M., Chu, B., Joa, J.H., Sang, M.K., et al. (2019). A preliminary examination of bacterial, archaeal, and fungal communities inhabiting different rhizocompartments of tomato plants under real-world environments. *Scientific reports* 9(1), 9300.

44. Li, H., Su, J.Q., Yang, X.R., and Zhu, Y.G. (2019). Distinct rhizosphere effect on active and total bacterial communities in paddy soils. *Science of The Total Environment* 649, 422-430.

45. Li, P., Ye, S., Liu, H., Pan, A., Ming, F., and Tang, X. (2018). Cultivation of drought-tolerant and insect-resistant rice affects soil bacterial, but not fungal, abundances and community structures. *Frontiers in Microbiology* 9, 1390.

46. Li, Z., Zu, C., Wang, C., Yang, J., Yu, H., and Wu, H. (2016). Different responses of rhizosphere and non-rhizosphere soil microbial communities to consecutive Piper nigrum L. monoculture. *Scientific reports* 6, 35825.

47. Liu, J., Ngoc Ha, V., Shen, Z., Zhu, H., Zhao, F., and Zhao, Z. (2018). Characteristics of bulk and rhizosphere soil microbial community in an ancient Platycladus orientalis forest. *Applied Soil Ecology* 132, 91-98.

48. Liu, J., Yao, Q., Li, Y., Zhang, W., Mi, G., Chen, X., et al. (2019). Continuous cropping of soybean alters the bulk and rhizospheric soil fungal communities in a Mollisol of Northeast PR China. *Land Degradation and Development* 30(14), 1725-1738.

49. Liu, J., Yu, Z., Yao, Q., Hu, X., Zhang, W., Mi, G., et al. (2017). Distinct soil bacterial communities in response to the cropping system in a Mollisol of northeast China. *Applied Soil Ecology* 119, 407-416.

50. Liu, Y., and Ludewig, U. (2019). Nitrogen‐dependent bacterial community shifts in root, rhizome and rhizosphere of nutrient‐efficient Miscanthus x giganteus from long‐term field trials. *GCB Bioenergy* 11(11), 1334-1347.

51. Lopes, L.D., Pereira, E.S.M.C., and Andreote, F.D. (2016). Bacterial abilities and adaptation toward the rhizosphere colonization. *Frontiers in Microbiology* 7, 1341.

52. Luo, J., Tao, Q., Jupa, R., Liu, Y., Wu, K., Song, Y., et al. (2019). Role of vertical transmission of shoot endophytes in root-associated microbiome assembly and heavy metal hyperaccumulation in sedum alfredii. *Environmental science and technology* 53(12), 6954-6963.

53. Luo, J., Tao, Q., Wu, K., Li, J., Qian, J., Liang, Y., et al. (2017). Structural and functional variability in root-associated bacterial microbiomes of Cd/Zn hyperaccumulator Sedum alfredii. *Applied microbiology and biotechnology* 101(21), 7961-7976.

54. Lupwayi, N.Z., May, W.E., Kanashiro, D.A., and Petri, R.M. (2018). Soil bacterial community responses to black medic cover crop and fertilizer N under no-till. *Applied Soil Ecology* 124, 95-103.

55. Ma, M., Du, H., Sun, T., An, S., Yang, G., and Wang, D. (2019). Characteristics of archaea and bacteria in rice rhizosphere along a mercury gradient. *Science of The Total Environment* 650(1), 1640-1651.

56. Mapelli, F., Marasco, R., Fusi, M., Scaglia, B., Tsiamis, G., Rolli, E., et al. (2018). The stage of soil development modulates rhizosphere effect along a High Arctic desert chronosequence. *The ISME Journal* 12(5), 1188-1198.

57. Mosqueira, M.J., Marasco, R., Fusi, M., Michoud, G., Merlino, G., Cherif, A., et al. (2019). Consistent bacterial selection by date palm root system across heterogeneous desert oasis agroecosystems. *Scientific reports* 9(1), 4033.

58. Nuccio, E.E., Anderson-Furgeson, J., Estera, K.Y., Pett-Ridge, J., de Valpine, P., Brodie, E.L., et al. (2016). Climate and edaphic controllers influence rhizosphere community assembly for a wild annual grass. *Ecology* 97(5), 1307-1318.

59. Oberholster, T., Vikram, S., Cowan, D., and Valverde, A. (2018). Key microbial taxa in the rhizosphere of sorghum and sunflower grown in crop rotation. *Science of The Total Environment* 624, 530-539.

60. Pascual, J., Blanco, S., Ramos, J.L., and van Dillewijn, P. (2018). Responses of bulk and rhizosphere soil microbial communities to thermoclimatic changes in a Mediterranean ecosystem. *Soil Biology and Biochemistry* 118, 130-144.

61. Pathan, S.I., Ceccherini, M.T., Hansen, M.A., Giagnoni, L., Ascher, J., Arenella, M., et al. (2015). Maize lines with different nitrogen use efficiency select bacterial communities with different β-glucosidase-encoding genes and glucosidase activity in the rhizosphere. *Biology and Fertility of Soils* 51(8), 995-1004.

62. Pham, H.N., Michalet, S., Bodillis, J., Nguyen, T.D., Nguyen, T.K.O., Le, T.P.Q., et al. (2017). Impact of metal stress on the production of secondary metabolites in Pteris vittata L. and associated rhizosphere bacterial communities. *Environmental Science and Pollution Research* 24(20), 16735-16750.

63. Praeg, N., Pauli, H., and Illmer, P. (2019). Microbial Diversity in Bulk and Rhizosphere Soil of Ranunculus glacialis Along a High-Alpine Altitudinal Gradient. *Frontiers in Microbiology* 10, 1429.

64. Qiao, Q., Wang, F., Zhang, J., Chen, Y., Zhang, C., Liu, G., et al. (2017). The Variation in the Rhizosphere Microbiome of Cotton with Soil Type, Genotype and Developmental Stage. *Scientific reports* 7(1), 3940.

65. Qiao, Q., Zhang, J., Ma, C., Wang, F., Chen, Y., Zhang, C., et al. (2019). Characterization and variation of the rhizosphere fungal community structure of cultivated tetraploid cotton. *PLoS One* 14(10), e0207903.

66. Qin, H., Brookes, P.C., Xu, J., and Feng, Y. (2014). Bacterial degradation of Aroclor 1242 in the mycorrhizosphere soils of zucchini (Cucurbita pepo L.) inoculated with arbuscular mycorrhizal fungi. *Environmental Science and Pollution Research* 21(22), 12790-12799.

67. Quoreshi, A.M., Suleiman, M.K., Kumar, V., Manuvel, A.J., Sivadasan, M.T., Islam, M.A., et al. (2019). Untangling the bacterial community composition and structure in selected Kuwait desert soils. *Applied Soil Ecology* 138, 1-9.

68. Robbins, C., Thiergart, T., Hacquard, S., Garrido-Oter, R., Gans, W., Peiter, E., et al. (2018). Root-associated bacterial and fungal community profiles of arabidopsis thaliana are robust across contrasting soil p levels. *Phytobiomes Journal* 2(1), 24-34.

69. Robertson-Albertyn, S., Alegria Terrazas, R., Balbirnie, K., Blank, M., Janiak, A., Szarejko, I., et al. (2017). Root hair mutations displace the barley rhizosphere microbiota. *Frontiers in plant science*, *8*, 1094.

70. Rojas, X., Guo, J., Leff, J.W., McNear, D.H., Jr., Fierer, N., and McCulley, R.L. (2016). Infection with a shoot-specific fungal endophyte (epichloe) alters tall fescue soil microbial communities. *Microbial ecology* 72(1), 197-206.

71. Shao, T., Gu, X., Zhu, T., Pan, X., Zhu, Y., Long, X., et al. (2019). Industrial crop Jerusalem artichoke restored coastal saline soil quality by reducing salt and increasing diversity of bacterial community. *Applied Soil Ecology* 138, 195-206.

72. Shen, Z., Wang, B., Zhu, J., Hu, H., Tao, C., Ou, Y., et al. (2019). Lime and ammonium carbonate fumigation coupled with bio-organic fertilizer application steered banana rhizosphere to assemble a unique microbiome against Panama disease. *Microbial Biotechnology* 12(3), 515-527.

73. Shenton, M., Iwamoto, C., Kurata, N., and Ikeo, K. (2016). Effect of wild and cultivated rice genotypes on rhizosphere bacterial community composition. *Rice* 9(1),1-11.

74. Shi, S., Nuccio, E., Herman, D.J., Rijkers, R., Estera, K., Li, J., et al. (2015). Successional trajectories of rhizosphere bacterial communities over consecutive seasons. *mBio* 6(4), e00746.

75. Singh, J., Silva, K.J.P., Fuchs, M., and Khan, A. (2019). Potential role of weather, soil and plant microbial communities in rapid decline of apple trees. *PLoS One* 14(3), e0213293.

76. Su, J., Ouyang, W., Hong, Y., Liao, D., Khan, S., and Li, H. (2016). Responses of endophytic and rhizospheric bacterial communities of salt marsh plant (Spartina alterniflora) to polycyclic aromatic hydrocarbons contamination. *Journal of Soils and Sediments* 16(2), 707-715.

77. Sun, J., Zhang, Q., Li, X., Zhou, B., and Wei, Q. (2017). Apple replant disorder of pingyitiancha rootstock is closely associated with rhizosphere fungal community development. *Journal of Phytopathology* 165(3), 162-173.

78. Sun, X., Zhou, Y., Tan, Y., Wu, Z., Lu, P., Zhang, G., et al. (2018). Restoration with pioneer plants changes soil properties and remodels the diversity and structure of bacterial communities in rhizosphere and bulk soil of copper mine tailings in Jiangxi Province, China. *Environmental Science and Pollution Research* 25(22), 22106-22119.

79. Szoboszlay, M., Nather, A., Liu, B., Carrillo, A., Castellanos, T., Smalla, K., et al. (2019). Contrasting microbial community responses to salinization and straw amendment in a semiarid bare soil and its wheat rhizosphere. *Scientific reports* 9(1), 9795.

80. Tan, Y., Cui, Y., Li, H., Kuang, A., Li, X., Wei, Y., et al. (2017). Rhizospheric soil and root endogenous fungal diversity and composition in response to continuous Panax notoginseng cropping practices. *Microbiological Research* 194, 10-19.

81. Thomas, F., and Cebron, A. (2016). Short-term rhizosphere effect on available carbon sources, phenanthrene degradation, and active microbiome in an aged-contaminated industrial soil. *Frontiers in Microbiology* 7, 92.

82. Truu, M., Ostonen, I., Preem, J.-K., Lõhmus, K., Nõlvak, H., Ligi, T., et al. (2017). Elevated air humidity changes soil bacterial community structure in the silver birch stand. *Frontiers in Microbiology* 8, 557.

83. Ullah, A., Akbar, A., Luo, Q., Khan, A.H., Manghwar, H., Shaban, M., et al. (2019). Microbiome diversity in cotton rhizosphere under normal and drought conditions. *Microbial ecology* 77(2), 429-439.

84. Uroz, S., Buee, M., Murat, C., Frey-Klett, P., and Martin, F. (2010). Pyrosequencing reveals a contrasted bacterial diversity between oak rhizosphere and surrounding soil. *Environmental microbiology reports* 2(2), 281-288.

85. Visioli, G., Sanangelantoni, A.M., Vamerali, T., Dal Cortivo, C., and Blandino, M. (2018). 16S rDNA profiling to reveal the influence of seed-applied biostimulants on the rhizosphere of young maize plants. *Molecules* 23(6),1461.

86. Wang, P., Marsh, E.L., Kruger, G., Lorenz, A., and Schachtman, D.P. (2020). Belowground microbial communities respond to water deficit and are shaped by decades of maize hybrid breeding. *Environmental microbiology* 22(3), 889-904.

87. Wang, Q., Jiang, X., Guan, D., Wei, D., Zhao, B., Ma, M., et al. (2018). Long-term fertilization changes bacterial diversity and bacterial communities in the maize rhizosphere of Chinese Mollisols. *Applied Soil Ecology* 125, 88-96.

88. Wattenburger, C.J., Halverson, L.J., and Hofmockel, K.S. (2019). Agricultural management affects root-associated microbiome recruitment over maize development. *Phytobiomes Journal* 3(4), 260-272.

89. Wei, X., Hu, Y., Razavi, B.S., Zhou, J., Shen, J., Nannipieri, P., et al. (2019). Rare taxa of alkaline phosphomonoesterase-harboring microorganisms mediate soil phosphorus mineralization. *Soil Biology and Biochemistry* 131, 62-70.

90. Winston, M.E., Hampton-Marcell, J., Zarraonaindia, I., Owens, S.M., Moreau, C.S., Gilbert, J.A., et al. (2014). Understanding cultivar-specificity and soil determinants of the cannabis microbiome. *PLoS One* 9(6), e99641.

91. Yamamoto, K., Shiwa, Y., Ishige, T., Sakamoto, H., Tanaka, K., Uchino, M., et al. (2018). Bacterial diversity associated with the rhizosphere and endosphere of two halophytes: glaux maritima and salicornia europaea. *Frontiers in Microbiology* 9, 2878.

92. Yang, Y., Wang, N., Guo, X., Zhang, Y., and Ye, B. (2017). Comparative analysis of bacterial community structure in the rhizosphere of maize by high-throughput pyrosequencing. *PLoS One* 12(5), e0178425.

93. Yergeau, E., Sanschagrin, S., Maynard, C., St-Arnaud, M., and Greer, C.W. (2014). Microbial expression profiles in the rhizosphere of willows depend on soil contamination. *The ISME Journal* 8(2), 344-358.

94. Yurgel, S.N., Douglas, G.M., Dusault, A., Percival, D., and Langille, M.G.I. (2018). Dissecting community structure in wild blueberry root and soil microbiome. *Frontiers in Microbiology* 9, 1187.

95. Zachow, C., Muller, H., Tilcher, R., and Berg, G. (2014). Differences between the rhizosphere microbiome of Beta vulgaris ssp. maritima-ancestor of all beet crops-and modern sugar beets. *Frontiers in Microbiology* 5, 415.

96. Zeng, M., Zhong, Y., Cai, S., and Diao, Y. (2018). Deciphering the bacterial composition in the rhizosphere of Baphicacanthus cusia (NeeS) Bremek. *Scientific reports* 8(1), 15831.

97. Zhang, B., Zhang, J., Liu, Y., Guo, Y., Shi, P., and Wei, G. (2018). Biogeography and ecological processes affecting root-associated bacterial communities in soybean fields across China. *Science of The Total Environment* 627, 20-27.

98. Zhang, X., Zhang, R., Gao, J., Wang, X., Fan, F., Ma, X., et al. (2017). Thirty-one years of rice-rice-green manure rotations shape the rhizosphere microbial community and enrich beneficial bacteria. *Soil Biology and Biochemistry* 104, 208-217.

99. Zheng, H., Wang, X., Chen, L., Wang, Z., Xia, Y., Zhang, Y., et al. (2018). Enhanced growth of halophyte plants in biochar-amended coastal soil: roles of nutrient availability and rhizosphere microbial modulation. *Plant, cell and environment* 41(3), 517-532.

100. Zhu, P., Wang, Y., Shi, T., Zhang, X., Huang, G., and Gong, J. (2018). Intertidal zonation affects diversity and functional potentials of bacteria in surface sediments: A case study of the Golden Bay mangrove, China. *Applied Soil Ecology* 130, 159-168.

101. Zhu, W., Chu, Y., Ding, C., Huang, Q., Zhang, B., Zhang, W., et al. (2015). Assessing bacterial communities in the rhizosphere of 8-year-old genetically modified poplar (Populus spp.). *Journal of Forestry Research* 27(4), 939-947.

R procedures:

install.packages('metafor')

library('metafor')

dat<-read.delim("C:/Users/Huaihai/Dropbox/ORNL Project/Tillage Meta-analysis/Metafor/Method/F2.csv",sep=",")

ef<-escalc(measure="ROM",m1i=MT,m2i=MC,sd1i=ST,sd2i=SC,n1i=NT,n2i=NC, data=dat,var.names=c("LRR","LRR_var"),digits=4)

M<-rma.mv(yi=LRR,V=LRR_var, random= ~1|S,struct="CS",method="REML",digits=4,data=ef)

res.R<-rma.mv(yi=LRR,V=LRR_var, random= ~1|S,struct="CS",method="REML",digits=4,data=ef)

res.F<-rma.mv(yi=LRR,V=LRR_var, digits=4,data=ef)

100 * (vcov(res.R)[1,1] - vcov(res.F)[1,1]) / vcov(res.R)[1,1]

par(mfrow=c(2,2))

funnel(res.R, main="Standard Error")

funnel(res.R, yaxis="vi", main="Sampling Variance")

funnel(res.R, yaxis="seinv", main="Inverse Standard Error")

funnel(res.R, yaxis="vinv", main="Inverse Sampling Variance")

dat<-read.delim("C:/Users/Huaihai/Dropbox/ORNL Project/Tillage Meta-analysis/Metafor/Method/F_1.csv",sep=",")

res.F<-rma.mv(yi=LRR,V=LRR_var, digits=4,data=dat)

par(mfrow=c(2,2))

funnel(res.F, main="Standard Error")

dat<-read.delim("C:/Users/Huaihai/Dropbox/ORNL Project/Tillage Meta-analysis/Metafor/Method/F_3.csv",sep=",")

res_F<-rma(yi,vi, digits=4,data=dat)

res

par(mfrow=c(2,2))

funnel(res, main="Standard Error")

funnel(res, yaxis="vi", main="Sampling Variance")

funnel(res, yaxis="seinv", main="Inverse Standard Error")

funnel(res, yaxis="vinv", main="Inverse Sampling Variance")

dat<-read.delim("C:/Users/Huaihai/Dropbox/ORNL Project/Tillage Meta-analysis/Metafor/Method/F_3.csv",sep=",")

res_F<-rma(yi,vi, digits=4,data=dat)

dat<-read.delim("C:/Users/Huaihai/Dropbox/ORNL Project/Tillage Meta-analysis/Metafor/Method/B_3.csv",sep=",")

res_B<-rma(yi,vi, digits=4,data=dat)

funnel(res_F, main="Standard Error")

funnel(res_F, yaxis="vi", main="Sampling Variance")

funnel(res_B, main="Standard Error")

funnel(res_B, yaxis="vi", main="Sampling Variance")
